# Detectability of and interference by major and minor hemoglobin variants using a new-generation ion-exchange HPLC system with two switchable analysis modes

**DOI:** 10.1016/j.plabm.2023.e00346

**Published:** 2023-12-09

**Authors:** Daisuke Manita, Shinji Ogino, Stefaan Marivoet, Masatsune Ogura

**Affiliations:** a Bioscience Division, Tosoh Corporation, Japan; b Tosoh Europe N.V., Belgium; c Department of Clinical Laboratory Technology, Faculty of Medical Science, Juntendo University, Japan; d Department of Metabolism and Endocrinology, Eastern Chiba Medical Center, Japan

**Keywords:** Hemoglobin A_1c_, Hemoglobin variant, High-performance liquid chromatography, GR01

## Abstract

**Objectives:**

High-performance liquid chromatography (HPLC) is commonly used to measure hemoglobin A_1c_ (HbA_1c_) levels and detect hemoglobin variants (Hb-Vars). HLC-723GR01 (GR01) is a new-generation automated ion-exchange HPLC system with two switchable analysis modes, namely short (30 s/test) and long modes (50 s/test). We evaluated the general performance of both analysis modes of GR01 for quantifying HbA_1c_ and detecting Hb-Vars.

**Design and methods:**

We evaluated the instrument's precision based on CLSI protocol EP-05-A3. A comparison of the two analysis modes of GR01 against the standard mode of HLC-723G11 was performed on 100 whole blood samples. The GR01 long mode was compared with affinity HPLC (AF-HPLC) for detecting common Hb-Vars (HbE, HbD, HbS, and HbC, >20 samples). To examine the detection capability for minor Hb-Vars, we analyzed 26 Hb-Vars using multiple analyzers, including both analysis modes of GR01.

**Results:**

Both modes of GR01 had within-laboratory coefficients of variation of ≤1.0 % from four samples with HbA_1c_ concentrations of 32–86 mmol/mol. Good correlation was observed between GR01 and HLC-723G11. The results for HbA_1c_ detection in the presence of the major variants revealed a strong correlation between the long mode of GR01 and AF-HPLC (r = 0.986–0.998), and the difference biases ranged 0.1–1.9 mmol/mol. In the long mode, only one variant had a difference bias exceeding 14 % [10 % (%NGSP)].

**Conclusion:**

The two analysis modes of GR01 were fast and had high accuracy and reproducibility, indicating their utility for routine clinical use in measuring HbA_1c_ samples with Hb-Vars.

## Highlights

• We developed HLC-723GR01 using CEX-HPLC with two analysis modes for HbA_1c_.
• Both modes of HLC-723GR01 exhibited rapid and high analytical performance.
• In the long mode, only one variant had a difference bias >14 % [10 % (%NGSP)].
• HLC-723GR01 can be useful for hemoglobin variant measurement in clinical setting.

## 1 Introduction

In 2021, more than 500 million people worldwide had diabetes mellitus, and future projections suggest that the absolute number of people with diabetes will increase by 46 % by 2045 [1]. Diabetes is characterized by elevated blood glucose concentrations, and the measurement of hemoglobin A_1c_ (HbA_1c_) is widely used in the diagnosis of diabetes in defined patient populations and the assessment of glycemic control in patients with diabetes [2]. Long-term prospective studies, in particular the Diabetes Control and Complications Trial, the UK Prospective Diabetes Study, and the Kumamoto Study, provided clear evidence that diabetic complications are directly related to the mean blood glucose level as measured by the HbA_1c_ concentration [[3], [4], [5]]. Weykamp et al. described HbA_1c_ as a valuable indicator of long-term glycemic control and defined specific treatment targets and decision limits, and the variable has been successfully standardized [6].

Clinical laboratories use highly accurate methods for HbA_1c_ testing based on several different methodologies, i.e., cation exchange (CEX) high-performance liquid chromatography (HPLC), affinity HPLC (AF-HPLC), capillary electrophoresis, immunoassay, and enzymatic methods. CEX-HPLC is a highly reliable method for HbA_1c_ analysis [7].

We developed HLC-723GR01 (GR01) based on CEX-HPLC with two analysis modes for HbA_1c_; a standard short mode (short mode) and a standard long mode (long mode). The short mode measures HbA_1c_ within 30 s and detects three major hemoglobin variants [Hb-Vars; hemoglobin D (HbD), hemoglobin S (HbS), and hemoglobin C (HbC)] as the H-Var peak and the other major variant [hemoglobin E (HbE)] as the P-HV peak (glycated HbE). The HbA_1c_ concentration cannot be quantified using the short mode if the H-Var peak is detected. By contrast, the long mode can measure HbA_1c_ within 50 s and separate the four major Hb-Vars into individual peaks (HbE, P-HV; HbD, D+; HbS, S+; and HbC, C+). When these peaks are detected in the long mode, the HbA_1c_ concentration is calculated considering each Hb-Var peak, and HbA_1c_ is reportable. These two modes are switchable using the same column and eluents.

In this study, we evaluated the results of the two analysis modes of GR01 using samples with and without Hb-Vars.

## 2 Materials and methods

### 2.1 Samples

The ethics committee of Eastern Chiba Medical Center (Chiba, Japan; No. 184–2022) and the Bioscience Division of Tosoh Corporation (Tokyo, Japan; 21-03, 22-03, 22-04) approved this study. At entry, written informed consent was obtained from all participants.

The 100 study patients (age: 61.9 ± 13.7 years, male/female: 62/38, fasting plasma glucose: 143.6 ± 57.3 mg/dL, medication: insulin 24 %, glinide or sulfonylurea 23 %, DPP-4 inhibitors 43 %, metformin 46 %, SGLT-2 inhibitor 52 %, imeglimin 3 %, α-glucosidase inhibitor 16 %, GLP-1 receptor agonist 11 %, thiazolidinediones 6 %) consisted of outpatients with type 1 diabetes (n = 8), type 2 diabetes (n = 71), and other diabetes (n = 9) [gestational diabetes (4) steroid diabetes (3) or pancreatic diabetes (2)] patients without diabetes (n = 12) who were undergoing treatment at Eastern Chiba Medical Center. Whole blood samples were collected using NaF/EDTA2Na test tubes (BD Vacutainer, 367933, Becton Dickinson & Co.).

To assess the effect of differences in blood collection tubes on anticoagulants, six patients without diabetes (HbA_1c_: 38.0 ± 2.6 mmol/mol) and three donors with diabetes (HbA_1c_: 72.7 ± 6.4 mmol/mol) were recruited among volunteers at the Tokyo Research Center of Tosoh Corporation (Kanagawa, Japan). Their samples were collected using different blood tubes [EDTA2K (BD Vacutainer, 367845, Becton Dickinson & Co., Franklin Lakes, NJ, USA), EDTA3K (BD Vacutainer, 367857, Becton Dickinson & Co.), NaF/EDTA2Na (BD Vacutainer, 367933, Becton Dickinson & Co.), 3.2 % sodium citrate (BD Vacutainer, 363083, Becton Dickinson & Co.), and heparin lithium (BD Vacutainer, 365901, Becton Dickinson & Co.)].

Hb-Var samples (HbE, HbD, HbS, HbC, and other rare variants) with HbA_1c_ levels measured by AF-HPLC measured by Premier Hb9210 (Trinity Biotech, Bray, Ireland) were obtained from the European Reference Laboratory for Glycohemoglobin (Winterswijk, The Netherlands). They were residual samples based on an opt-out or informed consent according to the regulations of each country. The minor Hb-Var samples were genetically tested at Fukuyama Medical Laboratory Co., Ltd. (Hiroshima, Japan) in accordance with the Ethical Guidelines for Genetic Testing.

All clinical investigations were conducted in accordance with the tenets of the Declaration of Helsinki.

### 2.2 HbA_1c_ analysis

HbA_1c_ was measured in the short and (30 s/test) and long modes (50 s/test) using GR01 (Tosoh, software ver. 1.04) and the standard mode using HLC-723G11 (G11, Tosoh, software ver. 3.08). Both GR01 and G11 use CEX-HPLC to separate hemoglobin fractions. Common Hb-Vars can be detected using GR01 in the short mode, but HbA_1c_ cannot be detected because of interference by Hb-Vars. Conversely, HbA_1c_ can be detected in the presence of common Hb-Vars using GR01 in the long mode.

The minor Hb-Var samples were also analyzed using the following assays: G11 (variant mode, 60 s/test), HLC-723GX (CEX-HPLC, variant mode, software ver. 1.24, 132 s/test), HLC-723G8 (CEX-HPLC, variant mode, software ver. 5.29, 90 s/test), HLC-723G8 (AF-HPLC, software ver. 5.29, affinity mode), and high-resolution HPLC based on the KO500 method [8].

All HbA_1c_ results based on CEX-HPLC were calculated using the total area excluding the hemoglobin F area.

HbA_1c_ values were expressed in the National Glycohemoglobin Standardization Program (NGSP) unit (%, one digit after the decimal point) using calibrators with NGSP units for GR01 evaluations. NGSP units were calculated from International Clinical Federation of Clinical Chemistry and Laboratory Medicine (IFCC) unit (mmol/mol, integer value) using the following formula: NGSP = [0.09148 * IFCC] + 2.152 [8].

### 2.3 Statistical analysis

Statistical analyses were performed using Analyse-it (Analyse-it Software, Ltd., Leeds, UK) with Excel (Microsoft Corp., Redmond, WA, USA).

## 3 Results

### 3.1 Precision

The precision of the assay was evaluated using the CLSI EP05-A3 protocol [9]. Level 1 (assignment value: 31 ± 3 mmol/mol, [5.0 % ± 0.3 %]) and Level 2 control samples (assignment value: 84 ± 5 mmol/mol [9.9 % ± 0.5 %]) from the Hemoglobin A_1c_ Control Set (Lot No AB0060, Tosoh) and two frozen patient samples (34 and 64 mmol/mol, respectively) were assayed in duplicate twice daily for 20 working days (20 × 2 × 2 experiment). Precision data for the short and long modes are summarized in Table 1. In the analysis, repeatability coefficients of variation (CVs) of 0.2%–0.7 % (mmol/mol) [0.1%–0.4 % (NGSP%)], between-run CVs of 0.0%–0.7 % (mmol/mol) [0.0–0.5 % (NGSP%)], between-day CVs of 0.0%–0.7 % (mmol/mol) [0.0%–0.6 % (NGSP%)], and within-laboratory CVs of 0.6%–1.0 % (mmol/mol) [0.4%–0.6 % (NGSP%)] were recorded.

**Table 1:** Precision testing of GR01 in the short and long modes.

	Short mode				Long mode			
	Control sample		Whole blood (frozen)		Control sample		Whole blood (frozen)	
	Level 1	Level 2	Sample 1	Sample 2	Level 1	Level 2	Sample 1	Sample 2
Mean mmol/mol	32.0	86.5	62.1	51.8	32.0	86.3	61.9	51.5
Repeatability CV (%)	0.3	0.4	0.4	0.2	0.5	0.3	0.2	0.7
Between-run CV (%)	0.5	0.3	0.4	0.7	0.5	0.4	0.4	0.0
Between-day CV (%)	0.0	0.4	0.5	0.3	0.0	0.5	0.4	0.7
Within-laboratory CV (%)	0.6	0.7	0.7	0.8	0.7	0.7	0.6	1.0
Mean % NGSP	5.10	10.05	7.81	6.88	5.10	10.03	7.79	6.85
Repeatability CV (%)	0.2	0.4	0.3	0.2	0.3	0.3	0.1	0.5
Between-run CV (%)	0.3	0.3	0.3	0.5	0.3	0.3	0.3	0.0
Between-day CV (%)	0.0	0.3	0.4	0.2	0.0	0.4	0.3	0.6
Within-laboratory CV (%)	0.4	0.6	0.6	0.6	0.4	0.6	0.5	0.8

Abbreviations: CV, Coefficients of variation; NGSP, National Glycohemoglobin Standardization Program.

### 3.2 Influence of anticoagulants

To assess whether anticoagulants affect the detection of HbA_1c_, blood was collected from donors with and without diabetes into different blood tubes [EDTA2K, EDTA3K, NaF/EDTA2Na (with or without centrifugation), 3.2 % sodium citrate, and heparin lithium]. The differences relative to EDTA2K tubes on day 0 were 97%–105 % for the short mode (Supplemental Table 1) and 100%–105 % for the long mode (Supplemental Table 2). No significant tube-dependent effects on HbA_1c_ measurements were observed using GR01 in the presence of any anticoagulant. The results also indicated that the stability of HbA_1c_ was not significantly altered in vials containing different anticoagulants after 15 days of storage at 4 °C.

### 3.3 Method comparison between GR01 and G11

**Fig. 1:**
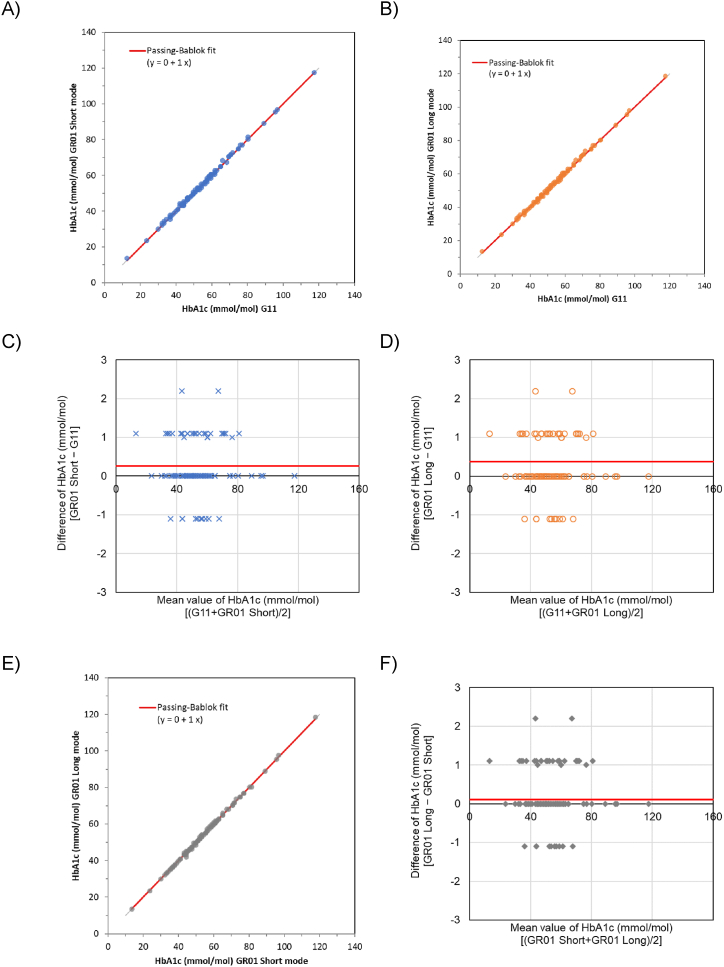
Method comparison between G11 and GR01. (A) Passing–Bablok regression plot. Y-axis, HbA_1c_ measured using the short mode of GR01 (mmol/mol); X-axis, standard mode of G11 (mmol/mol). y = 1.00 × + 0.00; r = 0.999. (B) Passing–Bablok regression plot. Y-axis, HbA_1c_ measured using the long mode of GR01 (mmol/mol); X-axis, standard mode of G11 (mmol/mol). y = 1.00 × + 0.00; r = 0.999. (C) Bland–Altman difference plot. Y-axis, difference value between the short mode of GR01 and the standard mode of G11 (mmol/mol); X-axis, mean of HbA_1c_ level (mmol/mol). Lines indicate the bias: 0.26 mmol/mol. (D) Bland–Altman difference plot. Y-axis, difference value between the long mode of GR01 and standard mode of G11 (mmol/mol); X-axis, mean HbA_1c_ level (mmol/mol). Lines indicate the bias: 0.37 mmol/mol. (E) Passing–Bablok regression plot. Y-axis, HbA_1c_ measured using the long mode of GR01 (mmol/mol); X-axis, short mode of GR01 (mmol/mol). y = 1.00 × + 0.00; r = 0.999. (F) Bland–Altman difference plot. Y-axis, difference value between the long and short modes of GR01 (mmol/mol); X-axis, mean HbA_1c_ level (mmol/mol). Lines indicate the bias: 0.11 mmol/mol.

A method comparison was performed using 100 fresh whole blood samples from subjects with and without diabetes according to the CLSI EP09-A3 method [10]. All results were reported without flags and with normal patterns of chromatograms. Passing–Bablok regression analysis of the data obtained using the short and long modes of GR01 displayed good correlations with the standard mode of G11 (r = 0.999, slope = 1.00, and intercept = 0.0 for both modes; Fig. 1-A and 1-B. Relative to G11, the Bland–Altman plot revealed mean difference biases of 0.26 and 0.37 mmol/mol for the short and long modes of GR01, respectively (Fig. 1-C and 1-D).

In addition, Passing–Bablok regression between the two modes of GR01 revealed a good correlation (r = 0.999) for HbA_1c_, as well as a slope of 1.00 and intercept of 0.00, and the Bland–Altman plot revealed a mean different bias of 0.11 mmol/mol (Fig. 1-E and 1-F).

### 3.4 Interference by common Hb-Vars

**Fig. 2:**
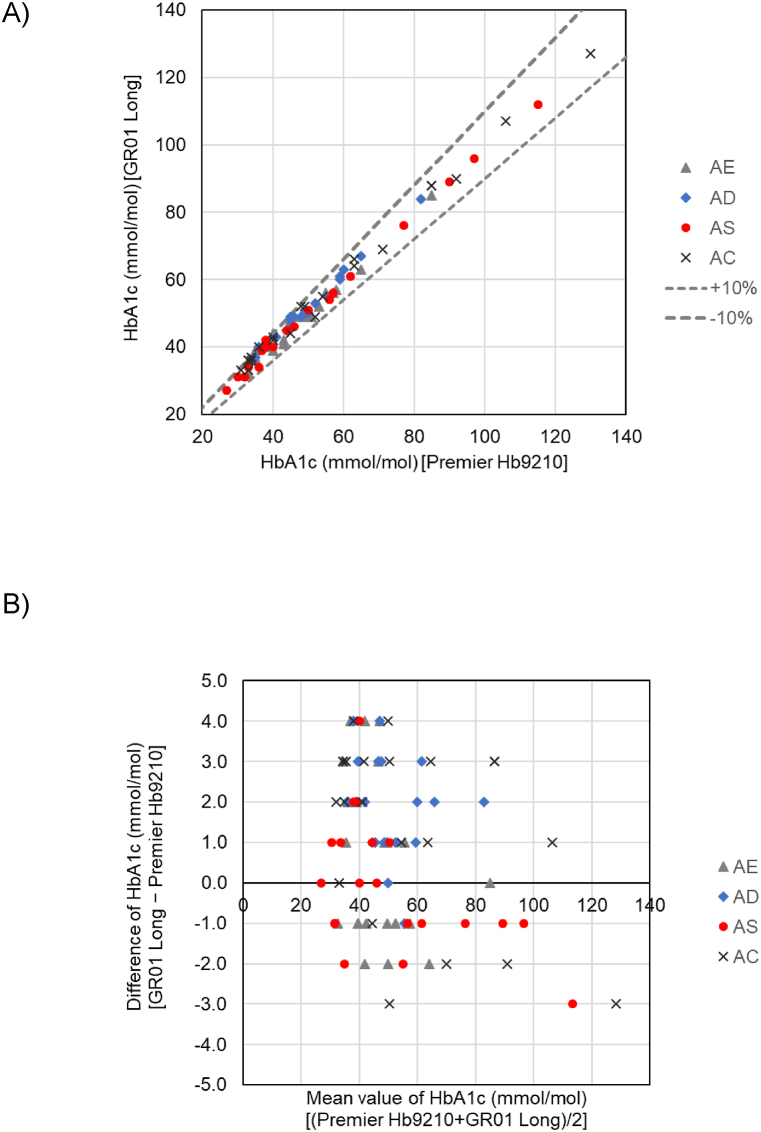
Interference by common Hb-Vars in the long mode of GR01. (A) Passing–Bablok non-parametric regression: long mode of GR01 vs. AF mode of Premier Hb9210. Y-axis. HbA_1c_ measured using the long mode of GR01 (mmol/mol); X-axis, HbA_1c_ measured using the AF mode of Premier Hb9210 (mmol/mol). Dotted lines indicate the range from 90 % to 110 % on the X-axis. (B) Bland–Altman difference plot. Y-axis, difference value of HbA_1c_ (mmol/mol); X-axis, mean HbA_1c_ level (mmol/mol).

To evaluate interference by common Hb-Vars in HbA_1c_ measurement, we performed a method comparison between the long mode of GR01 and the affinity mode of HLC-723G8 using 23 samples containing HbE, 22 samples containing HbD, 21 samples containing HbS, and 23 samples containing HbC. The results of Passing–Bablok regression and Bland–Altman plots are summarized in Table 2 and presented in Fig. 2.

**Table 2:** Long mode of GR01 mmol/mol (% NGSP).

Comparator	Samples	n	Passing–Bablok fit			Bias
			Slope	Intercept	r	
Affinity HPLC	HbE	23	0.943 (0.970)	2.982 (0.124)	0.986 (0.987)	0.5 (0.02)
	HbD	22	1.000 (1.000)	2.000 (0.100)	0.995 (0.995)	1.9 (0.15)
	HbS	21	0.968 (0.972)	1.484 (0.173)	0.998 (0.998)	0.1 (0.00)
	HbC	23	0.980 (1.000)	3.163 (0.200)	0.997 (0.997)	1.4 (0.13)

Good correlations were observed between the long mode of GR01 and affinity mode of HLC-723G8 using the aforementioned samples (r = 0.986–0.998) with a slope of 0.943–1.000 and intercept of 1.484–3.163.

### 3.5 Interference by minor Hb-Vars

We analyzed 26 types of minor Hb-Var and 4 types of common Hb-Var samples using the short and long modes of GR01 and affinity mode of HLC-723G8. The results are summarized in Table 3, and detailed results obtained using the variant and standard modes of G11, variant modes of HLC-723GX and HLC-723G8, and HPLC based on the KO500 method are shown in Supplementary Figs. 1–30. All Hb-Var samples were genetically analyzed by sequencing β-globin, α1-globin, α2-globin, and GAP-PCR (−3.7, anti 3.7, SEA, FIL). Six (20 %) minor Hb-Vars did not exhibit any flags in the short mode. Only two Hb-Var (7 %) had no flags, and there was no information regarding the detection of the Hb-Var sample even using the long mode. In addition, 16 long and 20 short mode samples had masked HbA_1c_ concentrations. The HbA_1c_ levels of one long mode sample (3 %) and three short mode samples (10 %) differed by more than 14 % (mmol/mol) [10 % (% NGSP)] from those of AF-HPLC (Table 3).

**Table 3:** Detection and interference of GR01 in the short and long modes using samples containing minor and common Hb-Vars.

	Short mode			Long mode			AF-HPLC
Hb-Vars (Supplemental Figure no.)	HbA_1c_ mmol/mol (% NGSP)	Flag^†^	Detection	HbA_1c_ mmol/mol (% NGSP)	Flag^†^	Detection	HbA_1c_ mmol/mol (%NGSP)
DD (1)	Unreportable	a, d	Yes	Unreportable	a, d, e (D+)	Yes	95 (10.8)
EE (2)	Unreportable	d, f, g	Yes	Unreportable	a, d, f, g	Yes	40 (5.8)
CS (3)	Unreportable	a, b, d, e, g	Yes	Unreportable	a, b, d, e (S+, C+), g, h	Yes	37 (5.6)
ES (4)	Unreportable	a, d, e, f, g, h	Yes	Unreportable	a, d, e (S+), f, g, h	Yes	46 (6.4)
SS (5)	Unreportable	a, d, e, g	Yes	Unreportable	a, d, e (C+), g	Yes	20 (4.0)
CC (6)	Unreportable	a, d, e, g	Yes	Unreportable	a, d, e (C+), g	Yes	22 (4.2)
J-Baltimore (7)	25^↓↓^ (4.4^↓↓^)	none	No	Unreportable	a, b	Yes	37 (5.5)
St. Anna (8)	Unreportable	e	Yes	65 (8.1)	e (D+)	Yes	71 (8.6)
O-Arab (9)	Unreportable	e	Yes	70 (8.6)	e (S+)	Yes	75 (9.0)
Pierre-Bénite (10)	Unreportable	a, d	Yes	Unreportable	a, b, d	Yes	41 (5.9)
Gouda (11)	37^↓^ (5.5^↓^)	none	No	37^↓^ (5.5^↓^)	none	No	43 (6.1)
Athens-GA (12)	67 (8.3)	none	No	Unreportable	a, b	Yes	63 (7.9)
Hamadan (13)	30^↓↓^ (4.9^↓↓^)	none	No	Unreportable	a, b	Yes	56 (7.3)
K-Ibadan (14)	Unreportable	d	Yes	Unreportable	a, b, d	Yes	46 (6.5)
Ethiopia (15)	Unreportable	e	Yes	91 (10.5)	e (D+)	Yes	91 (10.5)
Gorwihl (16)	Unreportable	d	Yes	Unreportable	a, d	Yes	79 (9.4)
Q-Iran (17)	Unreportable	e	Yes	49 (6.6)	a, e (C+)	Yes	51 (6.8)
Köln (18)	Unreportable	e	Yes	11 (3.2)	a, b	Yes	<18 (<3.8)
Hounslow (19)	Unreportable	a, b, f	Yes	Unreportable	e(D+), f, h	Yes	56 (7.3)
Riccarton (20)	Unreportable	f	Yes	48 (6.5)	f	Yes	46 (6.4)
G-Accra (21)	Unreportable	a, e	Yes	81^↑↑^ (9.6^↑↑^)	e (D+)	Yes	67 (8.3)
Ullevaal (22)	Unreportable	d, f	yes	Unreportable	d, f	yes	50 (6.8)
G-Philadelphia (23)	20^↓↓^ (4.0^↓↓^)	none	no	34 (5.3)	e (D+)	yes	33 (5.2)
Hb Melusine (24)	58^↓^ (7.4^↓^)	none	no	58^↓^ (7.4^↓^)	none	no	52 (6.9)
S&β^+^Thalassemia (25)	Unreportable	d, e, g	yes	Unreportable	a, d, e (S+), g	yes	58 (7.5)
β^0^-Thalassemia (26)	Unreportable	a, d, g	yes	Unreportable	a, d, g	yes	52 (6.9)
AE (27)	45 (6.2)	f	yes	45 (6.2)	f	yes	43 (6.1)
AD (28)	Unreportable	e	yes	34 (5.3)	e (D+)	yes	36 (5.4)
AS (29)	Unreportable	e	yes	39 (5.7)	e (S+)	yes	40 (5.8)
AC (30)	Unreportable	a, e	yes	39 (5.8)	a, e (C+)	yes	40 (5.8)

^†^ indicates flags of unknown peaks (reportable flags) (a), unknown high peak (unknown peak >20 %, unreportable flag) (b), TP low (TP < 300, reportable flag) (c), TP too low (TP < 1, unreportable flag) (d), Hb-Var detected (short: unreportable flag, long: reportable flag) (e), HbE suspected (reportable flag) (f) peak not detected (reportable flag) (g), and multiple Hb-Vars detected (unreportable flags) (h).

N.D. indicates no reportable HbA_1c_ data.

^↓^ indicates the result is underestimated by >10 % (mmol/mol) [7 % (% NGSP)] versus AF-HPLC.

^↓↓^ indicates the result is underestimated by >14 % (mmol/mol) [10 % (% NGSP)] versus AF-HPLC.

^↑↑^ indicates the result is overestimated by >14 % (mmol/mol) [10 % (% NGSP)] versus AF-HPLC.

The detailed data and chromatograms are presented in Supplemental Figs. 1–30.

## 4 Discussion

More than 1000 different Hb-Vars have been reported, and the prevalence or types of Hb-Vars differ by race, country, or region [11,12]. Some studies reported the rate of positive populations of Hb-Vars including thalassemia as follows: China, 1074/311,024 (0.35 %) [13]; India, 12,131/65,779 (18.4 %) [14]; United Arab Emirates, 545/6420 (8.5 %) [15]; and Thailand, 636/26,013 (2.4 %) [16].

Approximately 20 % of the variants produce clinical phenotypes such as hemolytic anemia, polycythemia, and methemoglobinemia, whereas the remaining 80 % cause no abnormal phenotype [17]. Therefore, many of the Hb-Vars themselves do not require routine clinical detection. By contrast, it is critical in HbA_1c_ testing to account for the presence of Hb-Vars because they sometimes interfere with HbA_1c_ assessment, and the manner and extent of interference depend on the measurement method [18,19]. In addition, some Hb-Vars inhibit red blood cell turnover [[20], [21], [22], [23]], and the HbA_1c_ results, regardless of the method of measurement, could deviate from the actual glucose levels.

Recently, Zechmeister et al. reported that HPLC provided more information about the presence of Hb-Vars than enzymatic assays. Analyzers used to measure HbA_1c_ must have high throughput for rapid testing and high resolution for clearer separation, which represent contradicting goals [24].

To meet these requirements, we developed the new-generation CEX-HPLC analyzer GR01 with two switchable analysis modes, namely short and long, using the same reagents and column. The liquid chromatographic method for HbA_1c_ measurement was developed by Trivelli et al., in 1971 [25], and the method has been significantly improved. Moreover, HPLC is a potent tool for evaluating Hb-Vars. However, HPLC should not be used as a standalone method for the definitive identification of Hb-Var because some minor Hb-Vars cannot be detected. The accuracy of HbA_1c_ in principal Hb-Var samples (HbE, HbD, HbS, and HbC) was previously evaluated using commercial methods [26,27]. The present study evaluated the performance of GR01 using samples with or without Hb-Vars. The long mode results of GR01 exhibited a good correlation with those of AF-HPLC. Therefore, it was considered that the long mode was free of interference from common Hb-Vars.

In this study, the precision values were consistent with the recommendations of Sacks et al. of within-laboratory CVs of less than 2 % [28].

HbA_1c_ levels measured by off-site immunoassay and enzymatic assays have been reported to be significantly lower than those measured by on-site HPLC using blood samples collected in NaF-containing blood collection tubes [29,30]. Subsequently, the Committee on Standardization of Laboratory Testing Related to Diabetes Mellitus of the Japan Diabetes Society recommended the use of EDTA-containing tubes for HbA_1c_ measurement using a layer of erythrocytes harvested by centrifugation [31]. We evaluated the effect of anticoagulants on HbA_1c_ measurements performed using GR01 and blood collection tubes. The results of HbA_1c_ measurements using either the short or long mode after 15 days were not significantly different from those of the reference EDTA2K tube immediately after blood collection. However, this study used a small number of samples, and the use or storage period of these blood collection tubes was not clarified. The choice of blood collection tubes should be based on the operator's manuals and other information according to the regulations of each country.

As previously mentioned, a large number of Hb-Vars exist globally, making it essential to detect them regardless of whether they interfere with HbA_1c_ measurements. Previous studies suggested that HbA_1c_ results should always be interpreted in the clinical context of the patient in case unexpected Hb-Vars are present [18,32].

We evaluated the ability of GR01 in the short and long modes to detect minor Hb-Vars using 26 samples. The results suggested that the presence of Hb-Gouda affected HbA_1c_ measurements, and the peak could not be clearly determined even by using the long mode. The flag rules need to be improved for the detection of Hb-Vars using a larger number of samples in the future.

This study had two limitations. First, this was a single-center and single-device study. It is desirable to conduct multicenter studies or review multiple evaluation results in the future. The other limitation was that the evaluation was performed using only one sample of each Hb-Var. Therefore, the chromatograms of the same Hb-Vars with different HbA_1c_ concentrations or with other conditions might be different from those in this report. The 26 types of rare variants used in this study represent only a small portion of the more than 1000 reported Hb-Vars. Therefore, it must be recognized that not all Hb-Vars can be detected, and false reports can occur.

In conclusion, GR01, which has two switchable standard analysis modes, has good reproducibility for rapid HbA_1c_ measurements. If the frequency of Hb-Vars is low, it might be efficient to use the short mode and retest with the long mode if Hb-Vars are detected using the short mode. However, some Hb-Vars could be missed using either the short or long mode, and caution should be exercised.

## Funding

None.

## Ethical approval

Eastern Chiba Medical Center (No. 184), the Bioscience Division of Tosoh Corporation (21-03, 22-03, 22-04).

## Data statement

The data that support the findings of this study are available from the corresponding author, DM, upon reasonable request.

## CRediT authorship contribution statement

**Daisuke Manita:** Data curation, Formal analysis, Funding acquisition, Investigation, Project administration, Validation, Writing – original draft. **Shinji Ogino:** Data curation, Writing – review & editing. **Stefaan Marivoet:** Data curation, Writing – review & editing. **Masatsune Ogura:** Data curation, Funding acquisition, Project administration, Resources, Supervision, Writing – review & editing.

## Declaration of competing interest

DM and SO are employees of Tosoh Corp (Japan).

SM is an employee of Tosoh Europe N.V. (Belgium).

## Data Availability

Data will be made available on request.

## References

[1] Sun H., Saeedi P., Karuranga S. (2022). IDF diabetes atlas: global, regional and country-level diabetes prevalence estimates for 2021 and projections for 2045. Diabetes Res. Clin. Pract..

[2] Sherwani S.I., Khan H.A., Ekhzaimy A. (2016). Significance of HbA1c test in diagnosis and prognosis of diabetic patients. Biomark. Insights.

[3] Nathan D.M., Genuth S., Diabetes Control and Complications Trial Research Group (1993). The effect of intensive treatment of diabetes on the development and progression of long-term complications in insulin-dependent diabetes mellitus. N. Engl. J. Med..

[4] Epidemiology of Diabetes Interventions and Complications (EDIC) Research Group (1999). Epidemiology of diabetes interventions and complications (EDIC). Design, implementation, and preliminary results of a long-term follow-up of the diabetes control and complications trial cohort. Diabetes Care.

[5] Ohkubo Y., Kishikawa H., Araki E. (1995). Intensive insulin therapy prevents the progression of diabetic microvascular complications in Japanese patients with non-insulin-dependent diabetes mellitus: a randomized prospective 6-year study. Diabetes Res. Clin. Pract..

[6] Weykamp C. (2013). HbA1c: a review of analytical and clinical aspects. Ann Lab Med.

[7] Gupta S., Jain U., Chauhan N. (2017). Laboratory diagnosis of HbA1c: a review. J. Nano Res..

[8] Hoelzel W., Weykamp C., Jeppsson J.O. (2004). IFCC reference system for measurement of hemoglobin A1c in human blood and the national standardization schemes in the United States, Japan, and Sweden: a method-comparison study. Clin. Chem..

[9] Edition A.G.-T. (2014).

[10] Clinical and Laboratory Standards Institute. Measurement Procedure Comparison and Bias Estimation Using Patient Samples; Approved Guideline. third ed. CLSI document EP09-A3.vol. 2013. Wayne, PA: Clinical and Laboratory Standards Institute.

[11] Giardine B., Borg J., Viennas E. (2014). Updates of the HbVar database of human hemoglobin variants and thalassemia mutations. Nucleic Acids Res..

[12] Ross Hardison DHKC, Patrinos GP, Wajcman H, et al. A database of human hemoglobin variants and thalassemia mutations, https://globin.bx.psu.edu/hbvar/menu.html.2022.

[13] Xu A., Chen W., Xie W. (2020). Hemoglobin variants in southern China: results obtained during the measurement of glycated hemoglobin in a large population. Clin. Chem. Lab. Med..

[14] Warghade S., Britto J., Haryan R. (2018). Prevalence of hemoglobin variants and hemoglobinopathies using cation-exchange high-performance liquid chromatography in central reference laboratory of India: a report of 65779 cases. J Lab Physicians.

[15] Kamel K., Chandy R., Mousa H. (1980). Blood groups and types, hemoglobin variants, and G‐6‐PD deficiency among Abu Dhabians in the United Arab Emirates. Am. J. Phys. Anthropol..

[16] Srivorakun H., Singha K., Fucharoen G. (2014). A large cohort of hemoglobin variants in Thailand: molecular epidemiological study and diagnostic consideration. PLoS One.

[17] Miyazaki A., Kohzuma T., Kasayama S. (2012). Classification of variant forms of haemoglobin according to the ratio of glycated haemoglobin to glycated albumin. Ann. Clin. Biochem..

[18] Little R.R., Roberts W.L. (2009). A review of variant hemoglobins interfering with hemoglobin A1c measurement. J. Diabetes Sci. Technol..

[19] Schnedl W.J., Krause R., Halwachs-Baumann G. (2000). Evaluation of HbA1c determination methods in patients with hemoglobinopathies. Diabetes Care.

[20] Bry L., Chen P.C., Sacks D.B. (2001). Effects of hemoglobin variants and chemically modified derivatives on assays for glycohemoglobin. Clin. Chem..

[21] Chernoff A.I. (1958). The hemoglobin D syndromes. Blood.

[22] Mccurdy P.R. (1969). 32-DFP and 51-Cr for measurement of red cell life span in abnormal hemoglobin syndromes. Blood.

[23] Prindle K.H., Mccurdy P.R. (1970). Red cell lifespan in hemoglobin C disorders (with special reference to hemoglobin C trait). Blood.

[24] Zechmeister B., Erden T., Kreutzig B. (2022). Analytical interference of 33 different hemoglobin variants on HbA1c measurements comparing high-performance liquid chromatography with whole blood enzymatic assay: a multi-center study. Clin. Chim. Acta.

[25] Trivelli L.A., Ranney H.M., Lai H.T. (1971). Hemoglobin components in patients with diabetes mellitus. N. Engl. J. Med..

[26] Rohlfing C., Hanson S., Estey M.P. (2021). Evaluation of interference from hemoglobin C, D, E and S traits on measurements of hemoglobin A1c by fifteen methods. Clin. Chim. Acta.

[27] Lenters-Westra E., English E. (2022). The never ending story of Hb-variants interferences on the measurement of HbA1c. Clin. Chim. Acta.

[28] Sacks D.B., Bruns D.E., Goldstein D.E. (2002). Guidelines and recommendations for laboratory analysis in the diagnosis and management of diabetes mellitus. Clin. Chem..

[29] Koga M., Okuda M., Inada S. (2020). HbA1c levels measured by enzymatic assay during off-site health checkups are lower than those measured by on-site HPLC assay. Diabetol Int.

[30] Goya K., Tanaka S., Makio T. (2021). HbA1c levels measured by enzymatic assay and immunoassay during off-site health checkups are both lower than those measured by on-site HPLC Assay. Kobe J. Med. Sci..

[31] Kuwa K., Okahashi M., Sato A. (2021). Recommendation of the use of EDTA-containing tubes for the measurement of HbA1c using a layer of erythrocytes obtained after centrifugation. J. Jpn. Diabetes Soc..

[32] Sacks D.B. (2011). A1C versus glucose testing: a comparison. Diabetes Care.

