# Supplementary material for: Detectability of and interference by major and minor hemoglobin variants using a new-generation ion-exchange HPLC system with two switchable analysis modes
Source: Pract Lab Med. 2023 Dec 9;38:e00346. doi: 10.1016/j.plabm.2023.e00346 (PMC10716769; doi:10.1016/j.plabm.2023.e00346)
Supplement: Multimedia component 1 [file mmc1.docx]

Supplemental Table 1. Comparison of the blood test tubes with anticoagulants. The table shows the %differences of HbA1c using Standard Short mode for 0, 1, 2, 7, and 15 days vs 0day of EDTA 2K.

Supplemental Table 2. Comparison of the blood test tubes with anticoagulants. The table shows the %differences of HbA1c using Standard Long mode for 0, 1, 2, 7, and 15 days vs 0day of EDTA 2K.

C)

B)

A)


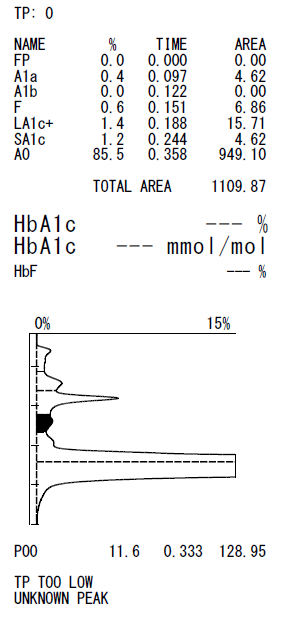

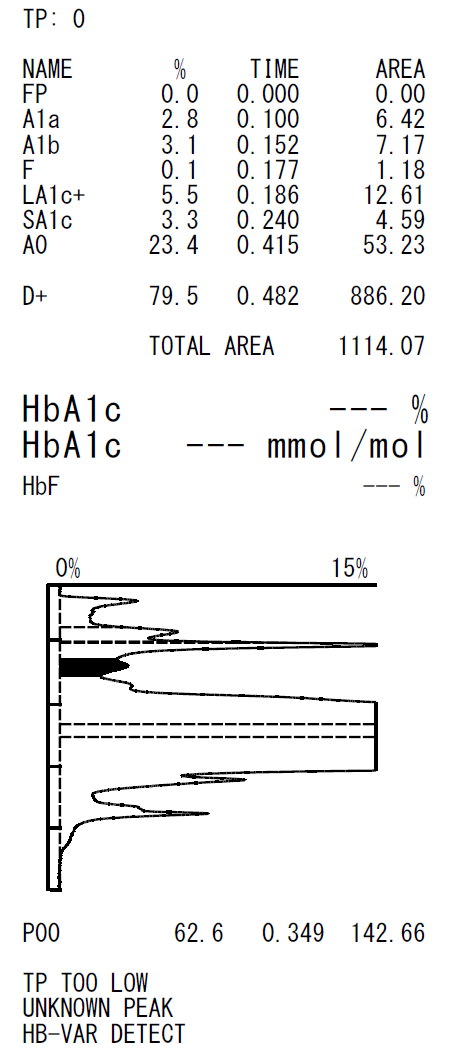

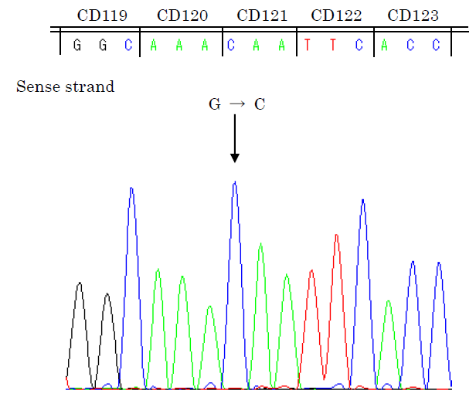


G)

D)

E)

F)


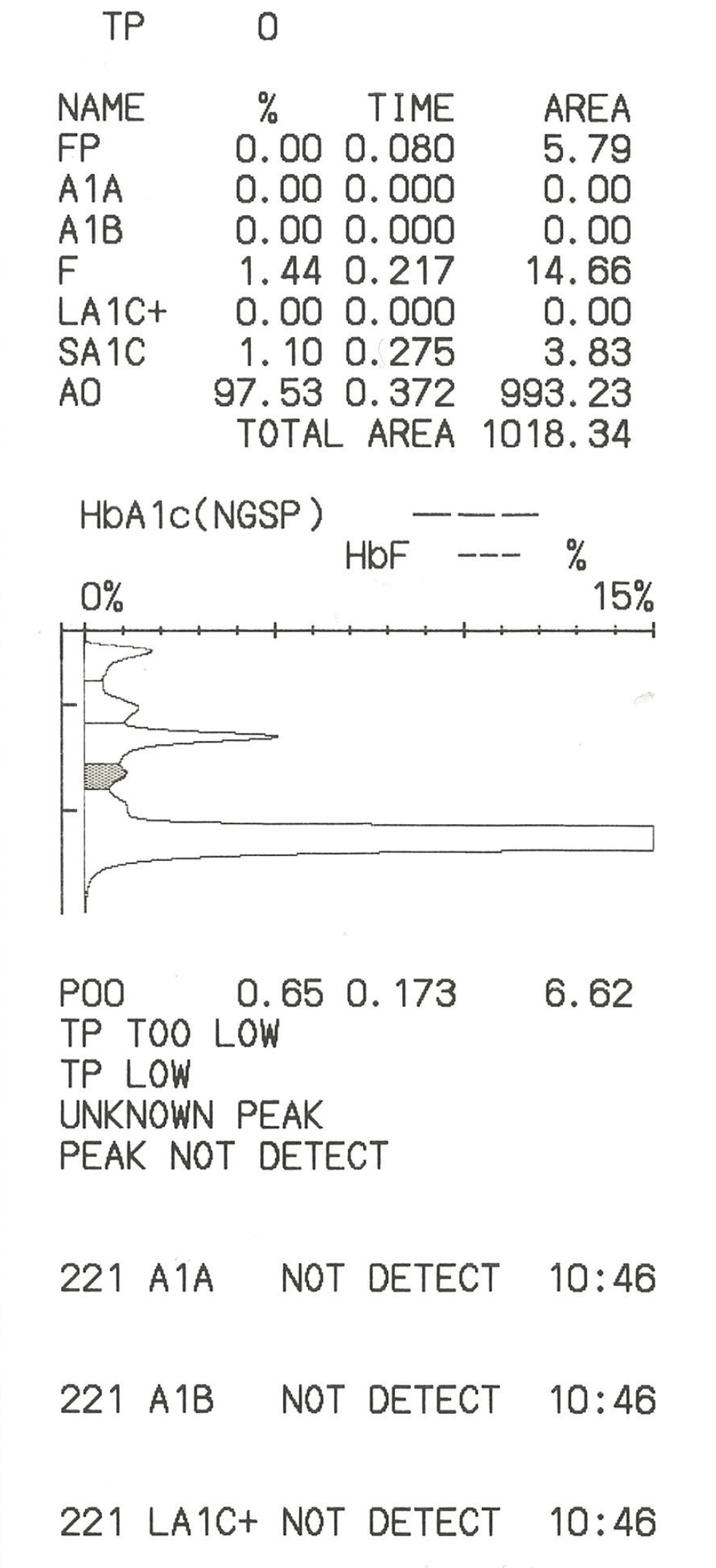

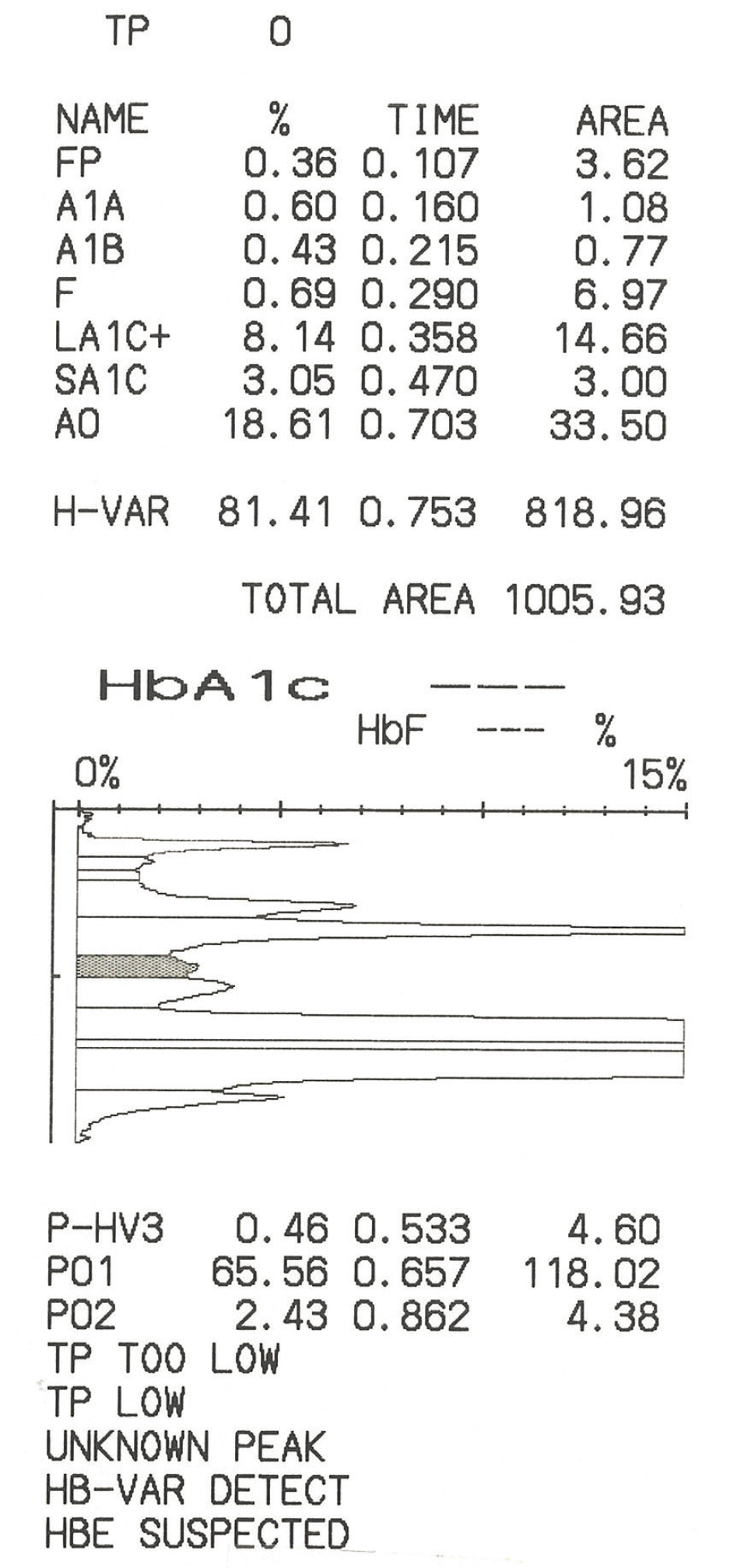

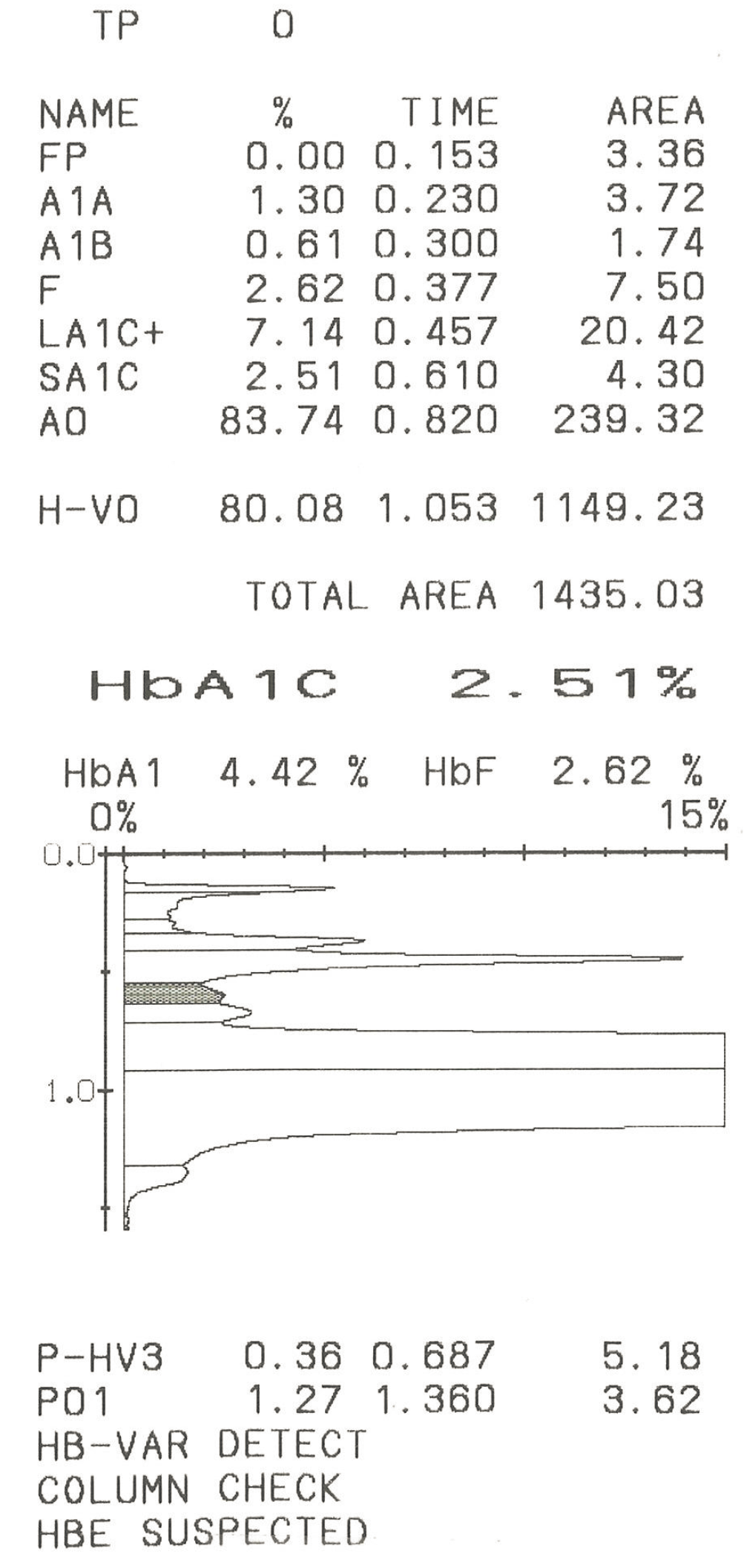

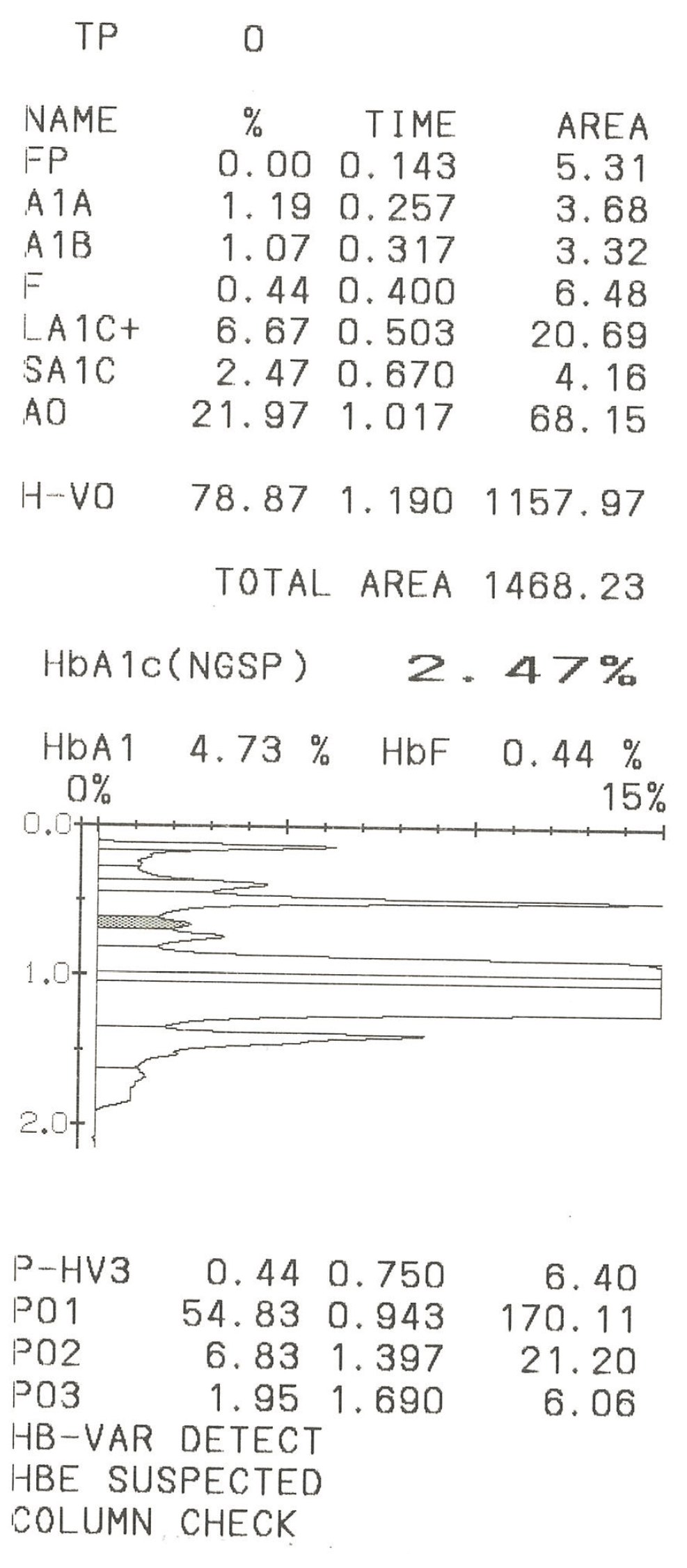


I)

H)


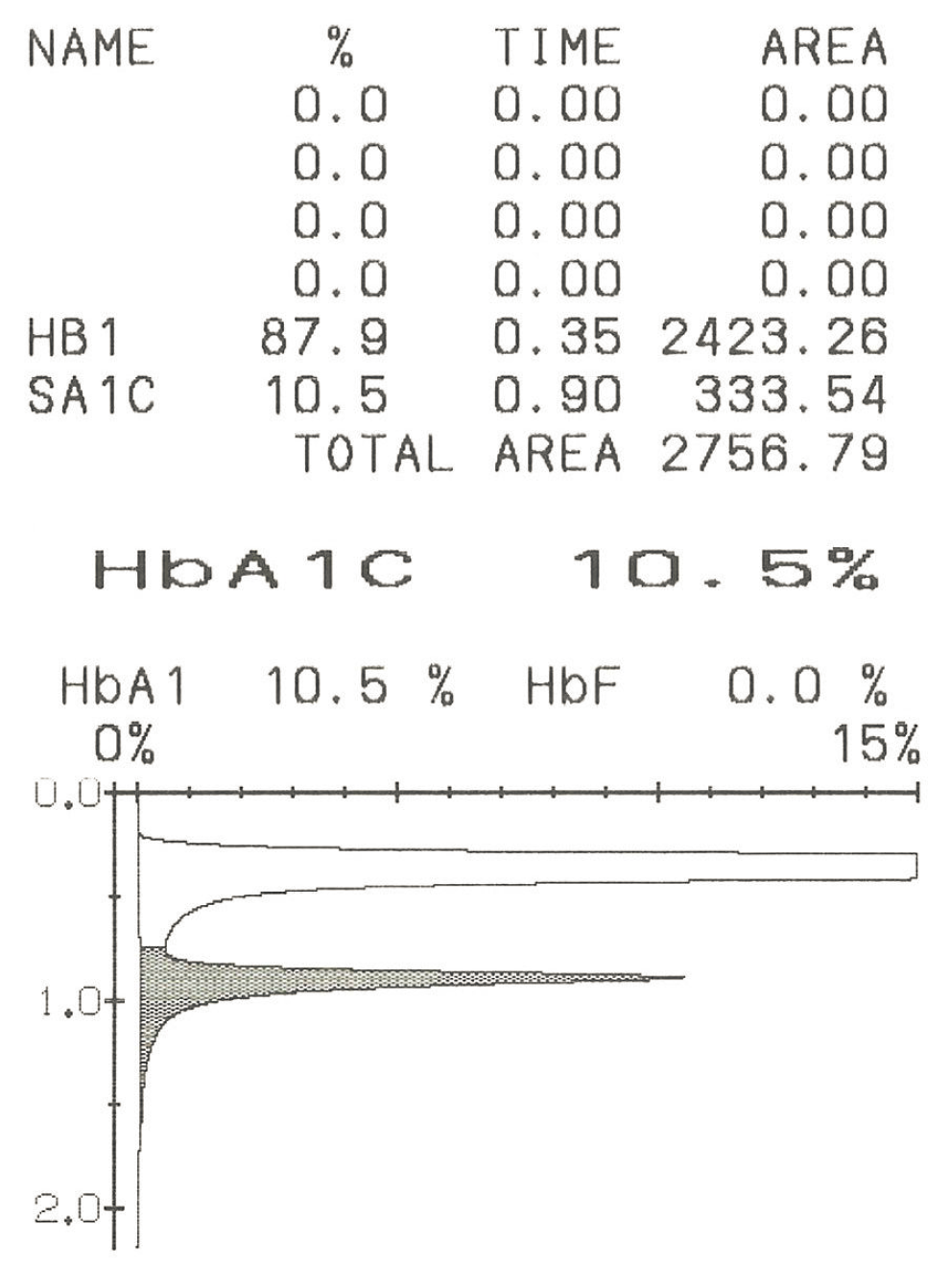

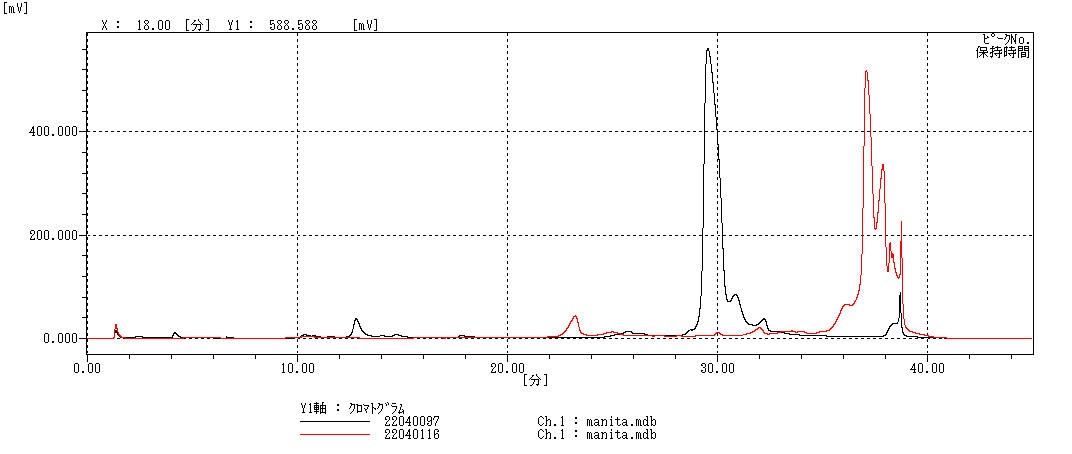


Supplemental Figure 1

Results of HbD-Los Angeles (homozygote or hemizygote) (Sample No. 600-11-01308-3).

(A) Results of the globin gene analysis. The mutation was identified at position 121 [from GAA (Glu) to CAA (Gln)].

Chromatograms obtained using HPLC methods; GR01-Short (B), GR01-Long (C), G11-Standard (D), G11-Variant (E), GX (F), G8-Variant (G) and G8-AF (H). (I) Chromatograms of HPLC system based on KO500 (Red: Variant sample, Black: control sample).

C)

B)

A)


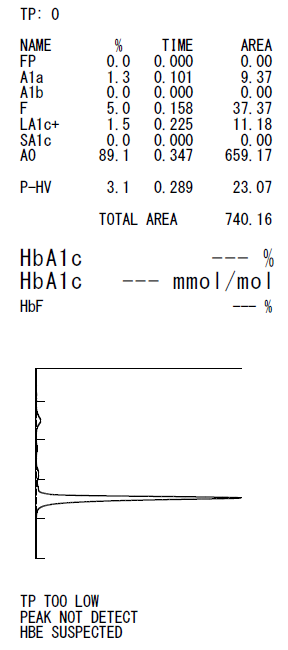

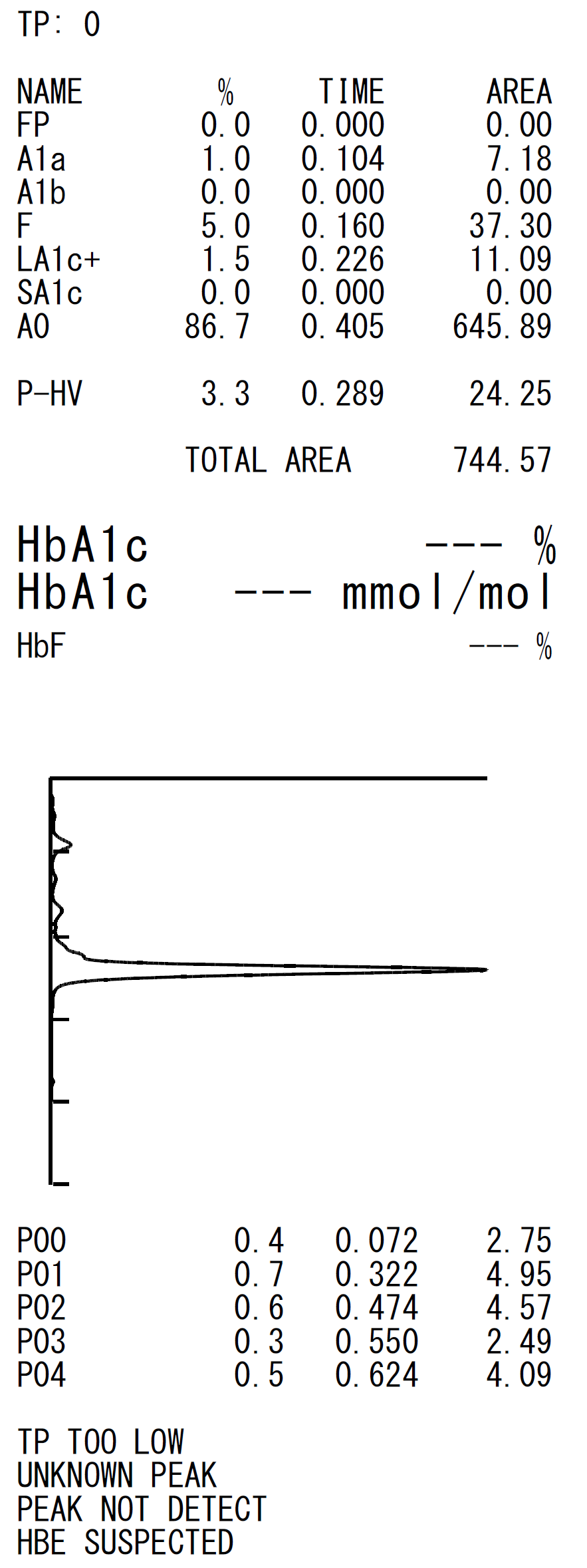

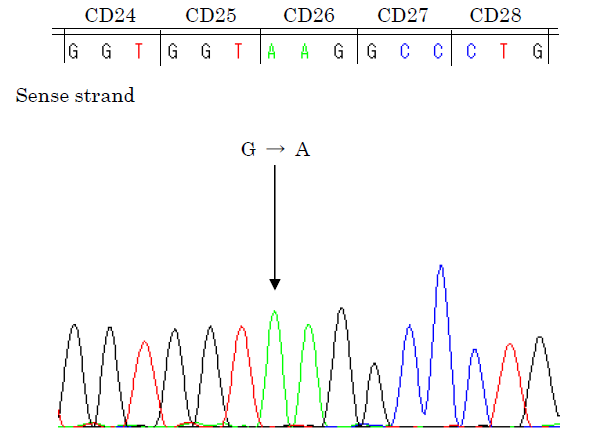


G)

D)

E)

F)


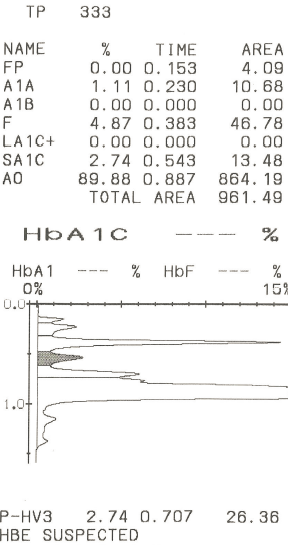

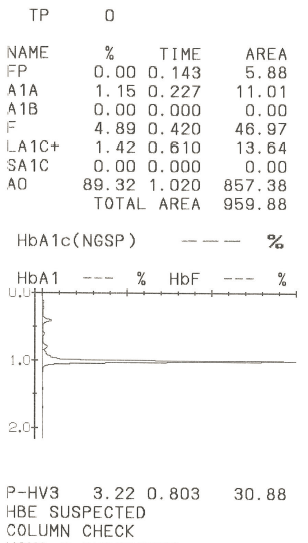

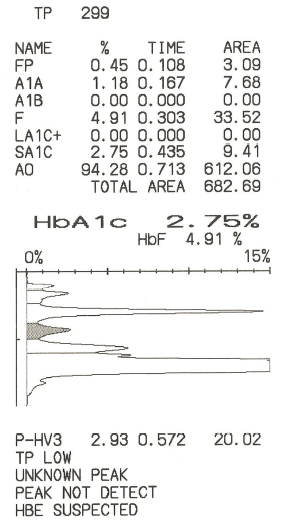

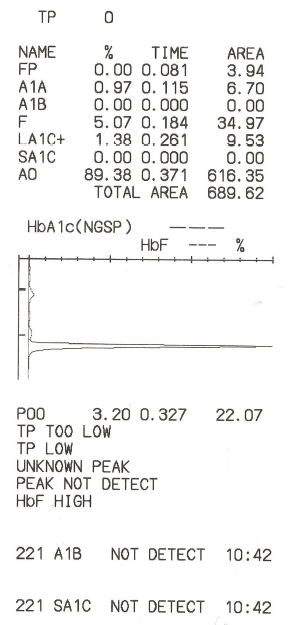


I)

H)


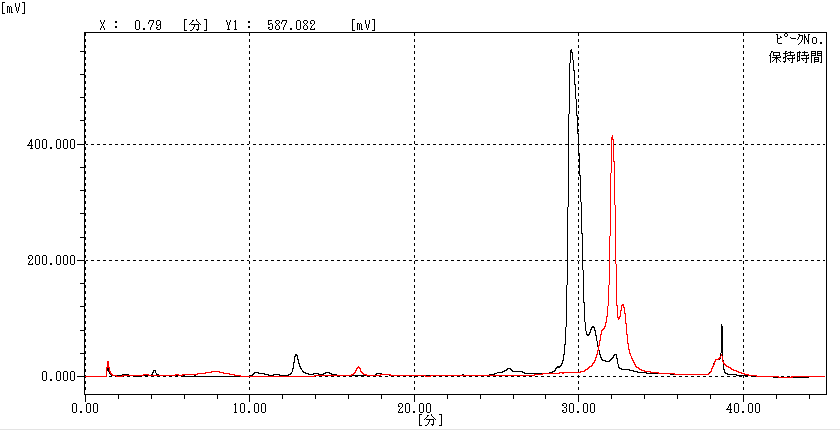

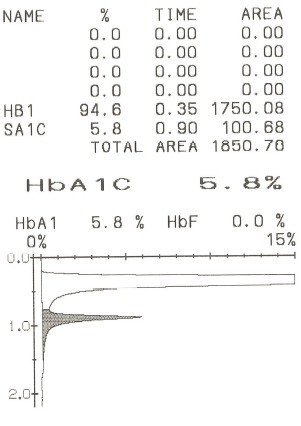


Supplemental Figure 2

Results of Hb E (homozygote or hemizygote) (Sample No. 600-17-9994-31).

(A) Results of the globin gene analysis. The mutation was identified at position 26 [from GAG (Glu) to AAG (Lys)] with SEA(+). Chromatograms obtained using HPLC methods; (B) GR01-Short, (C) GR01-Long, (D) G11-Standard, (E) G11-Variant, (F) GX, (G) G8-Variant, and (H) G8-AF. (I) Chromatograms of HPLC system based on KO500 (Red: Variant sample, Black: control sample).


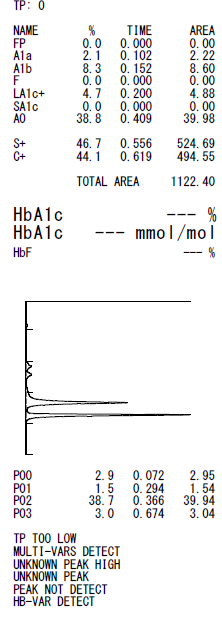


C)

B)

A)


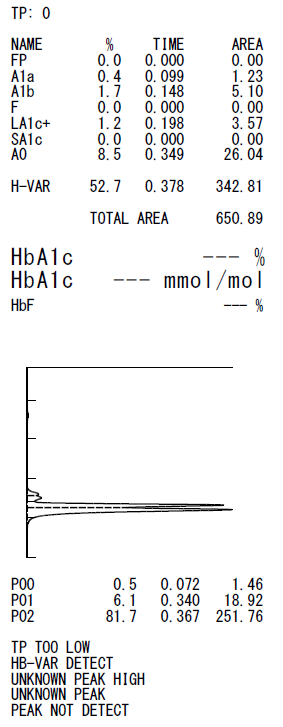

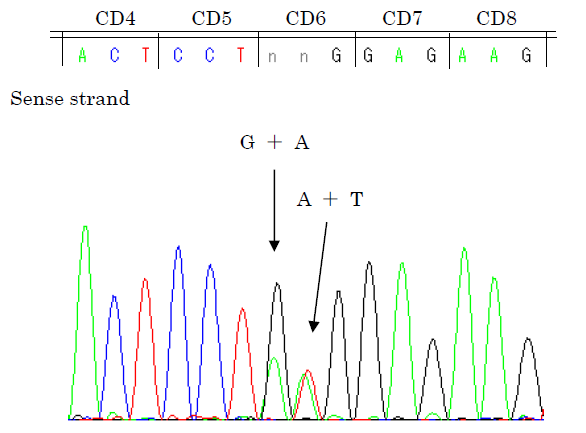


G)

D)

E)

F)


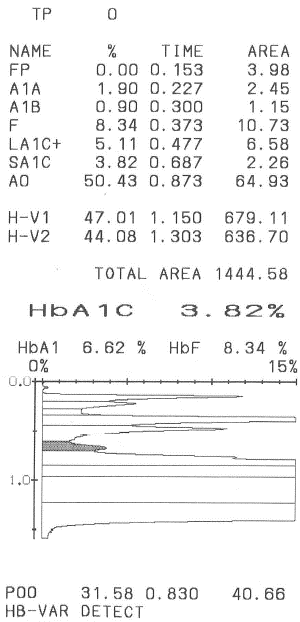

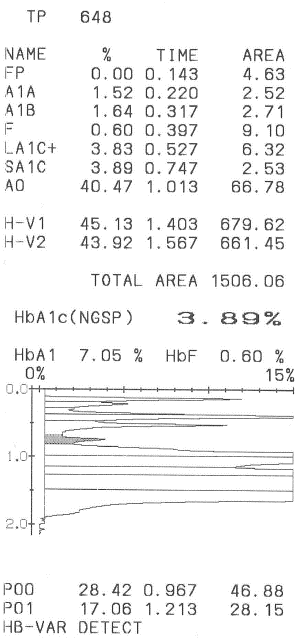

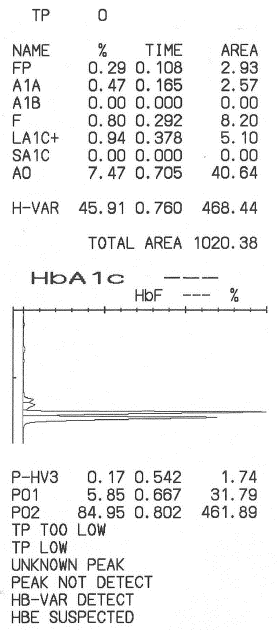

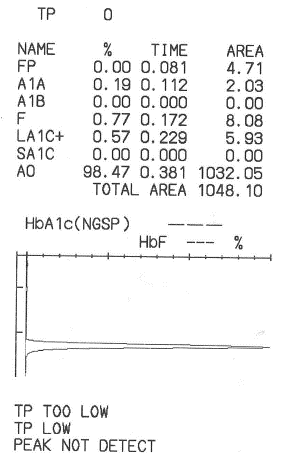


I)

H)


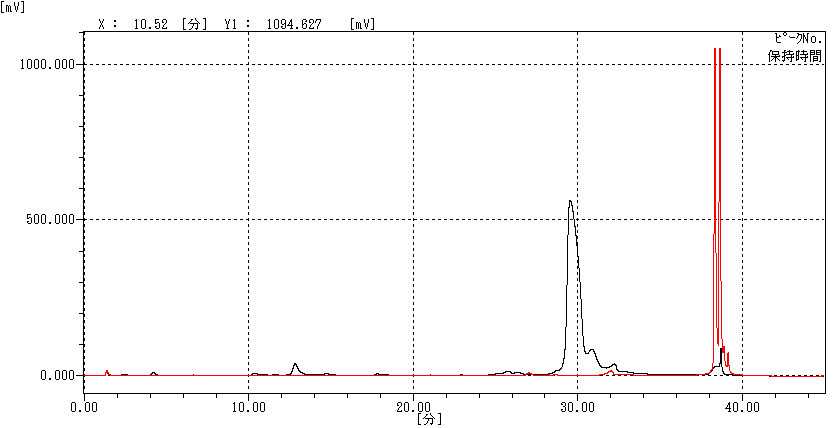

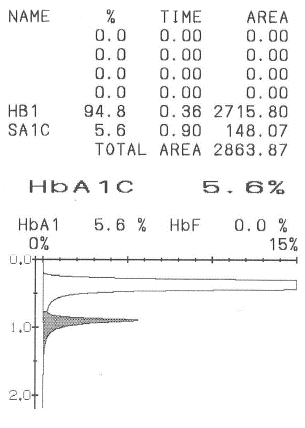


Supplemental Figure 3

Results of Hb C (heterozygote) and Hb S (heterozygote) (Sample No. 600-12-1314-27).

(A) Results of the globin gene analysis. The mutation was identified at position 6 [from GAG (Glu) to AAG (Lys)] and position 6 [from GAG (Glu) to GTG (Val)]. Chromatograms obtained using HPLC methods; (B) GR01-Short, (C) GR01-Long, (D) G11-Standard, (E) G11-Variant, (F) GX, (G) G8-Variant, and (H) G8-AF. (I) Chromatograms of HPLC system based on KO500 (Red: Variant sample, Black: control sample).


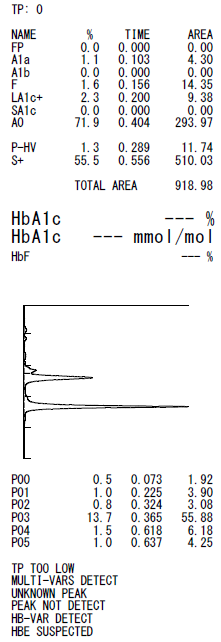


C)

B)

A)


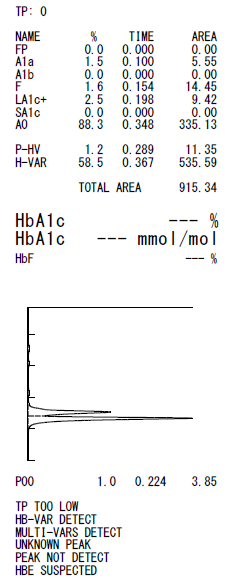

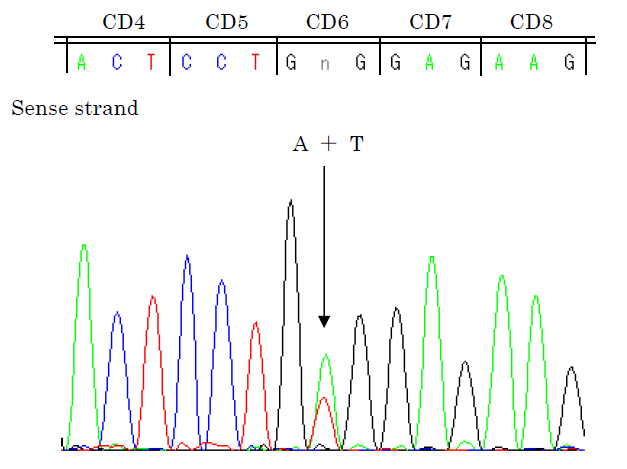


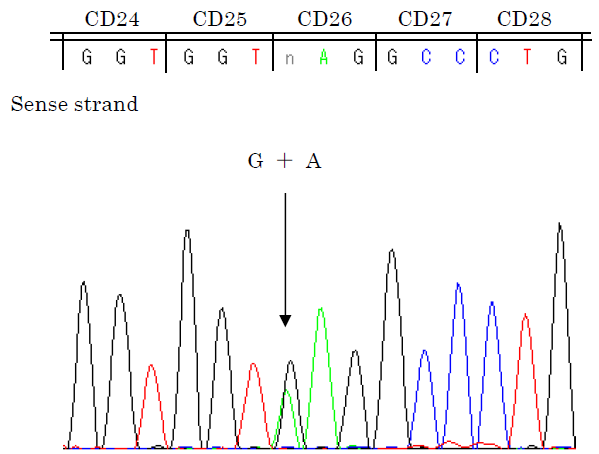


G)

D)

E)

F)


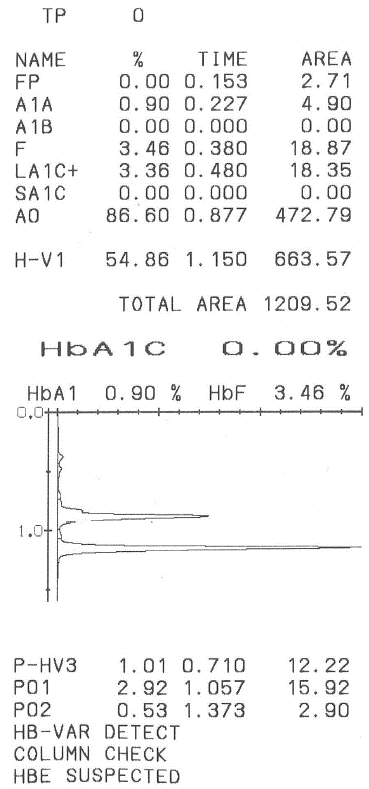

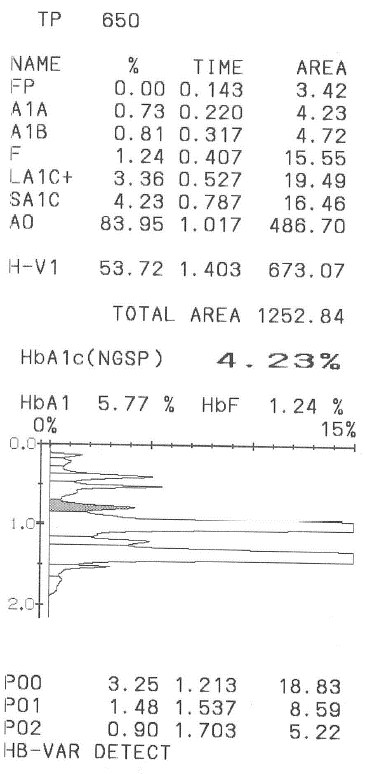

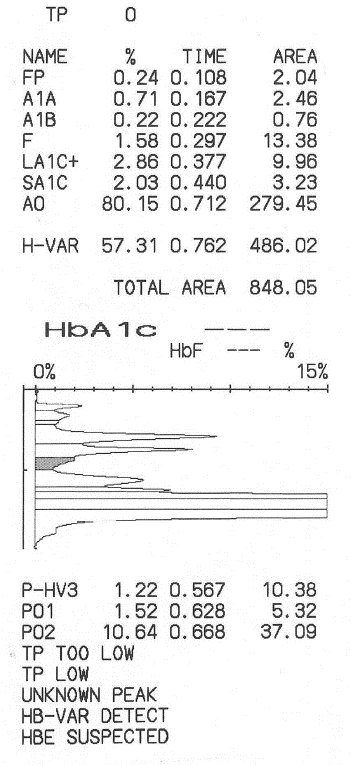

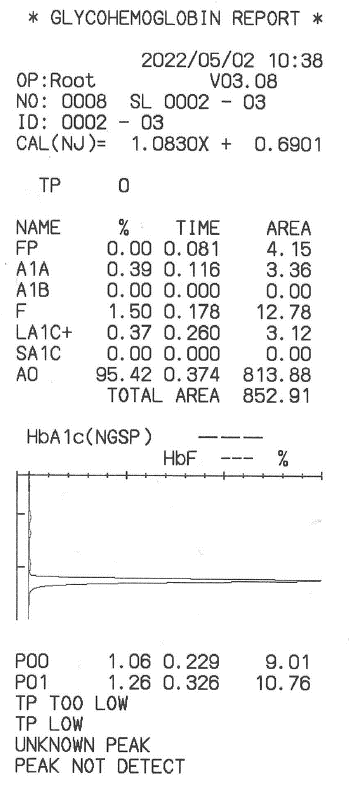


I)

H)


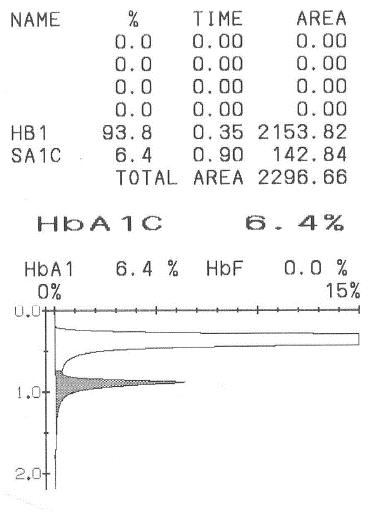

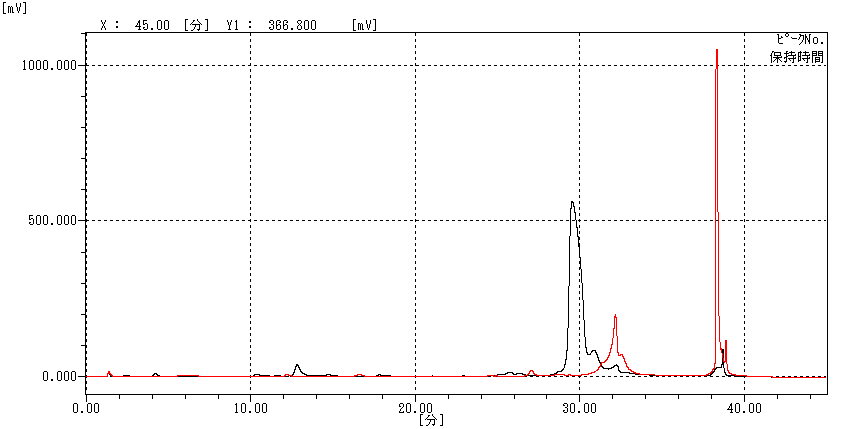


Supplemental Figure 4

Results of Hb E (heterozygote) and Hb S (heterozygote) (Sample No. 600-12-1313-26).

(A) Results of the globin gene analysis. The mutation was identified at position 6 [from GAG (Glu) to GTG (Val)] and position 26 [from GAG (Glu) to AAG (Lys)], and anti 3.7(+). Chromatograms obtained using HPLC methods; (B) GR01-Short, (C) GR01-Long, (D) G11-Standard, (E) G11-Variant, (F) GX, (G) G8-Variant, and (H) G8-AF. (I) Chromatograms of HPLC system based on KO500 (Red: Variant sample, Black: control sample).


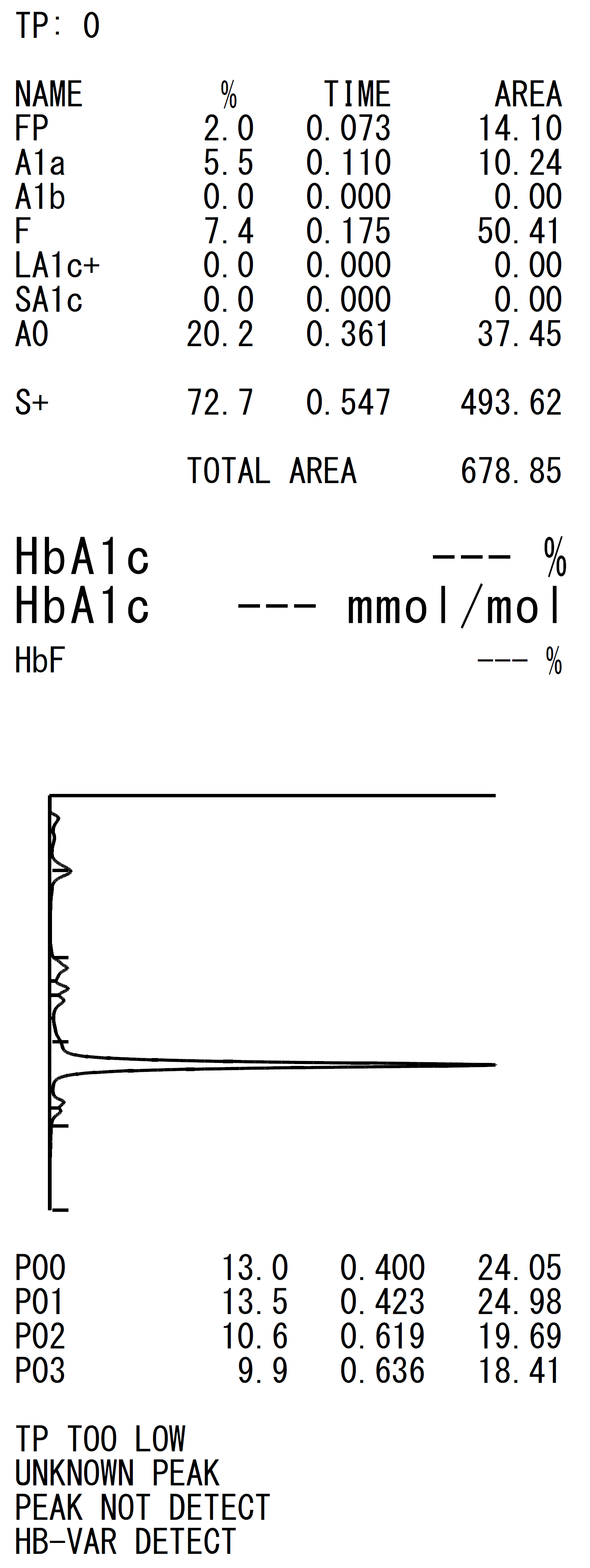


C)

B)

A)


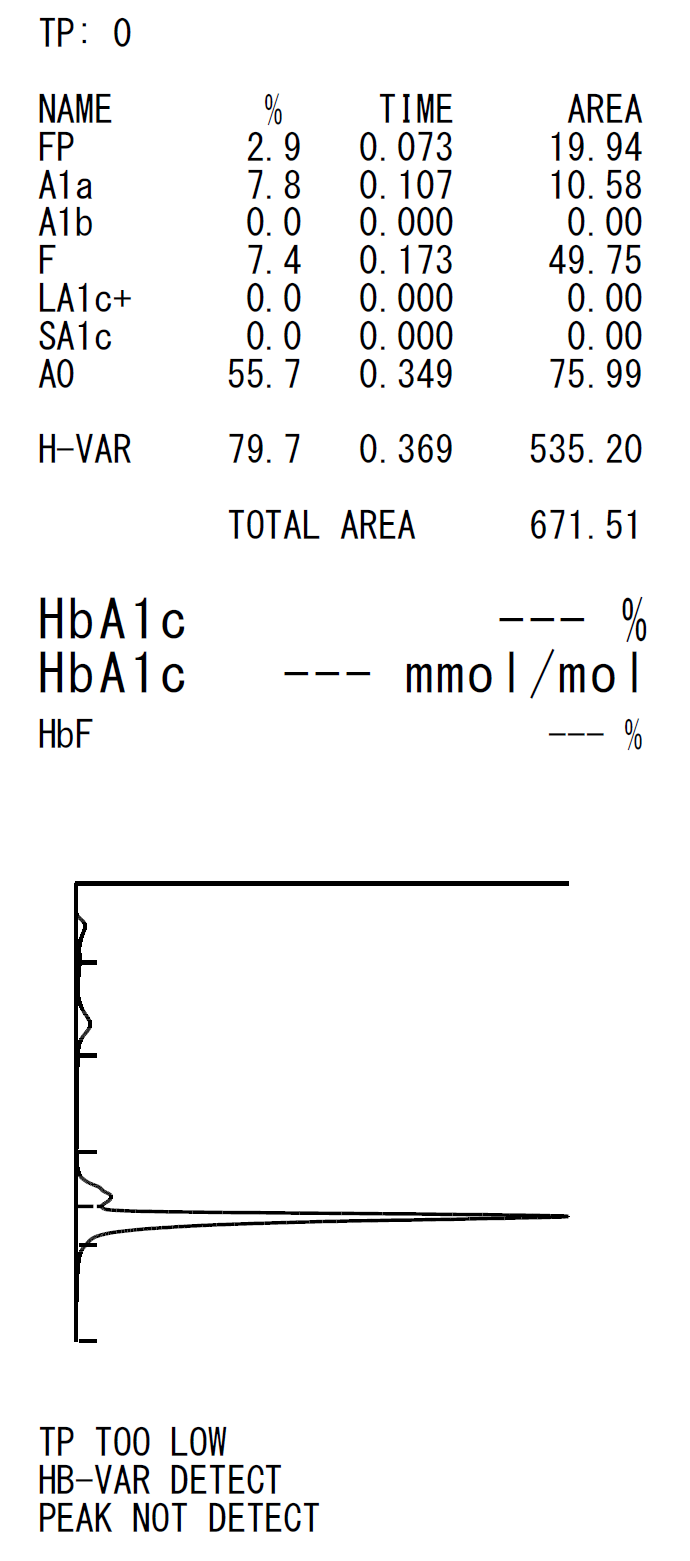

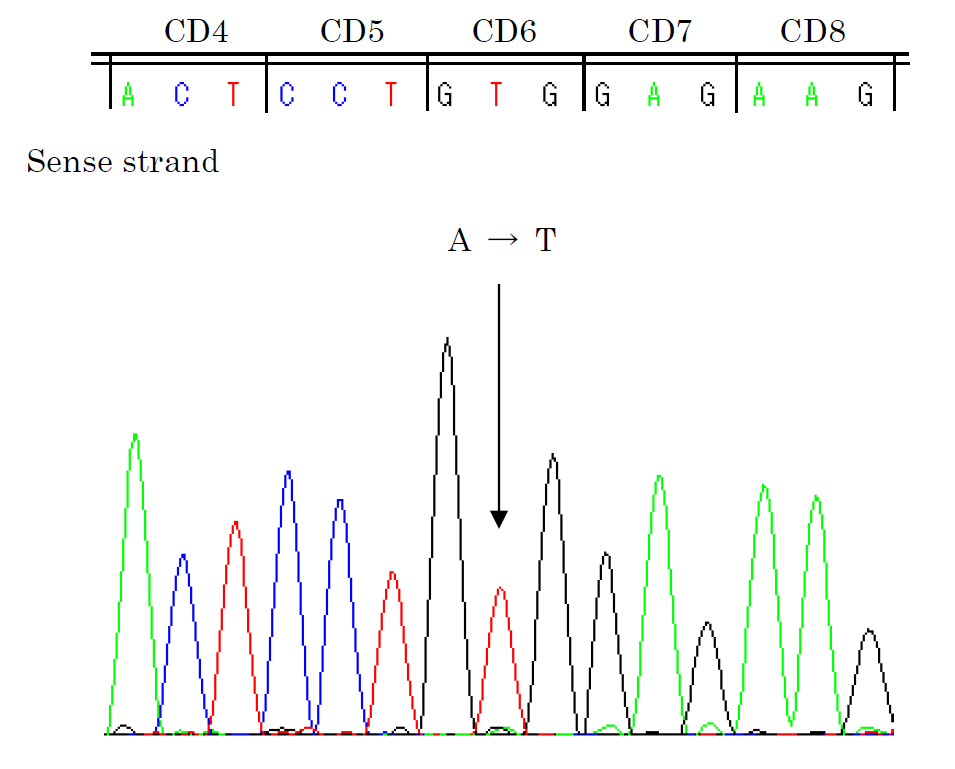


G)

D)

E)

F)


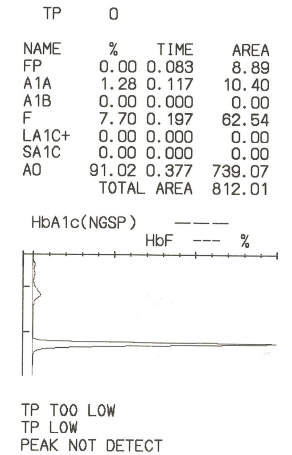

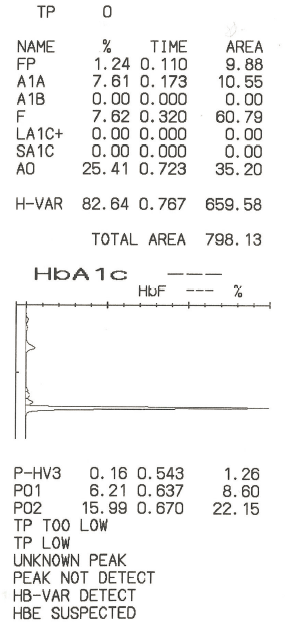

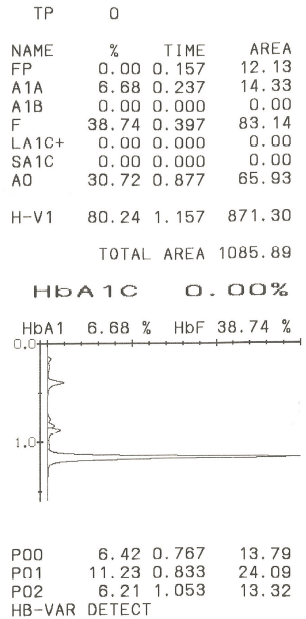

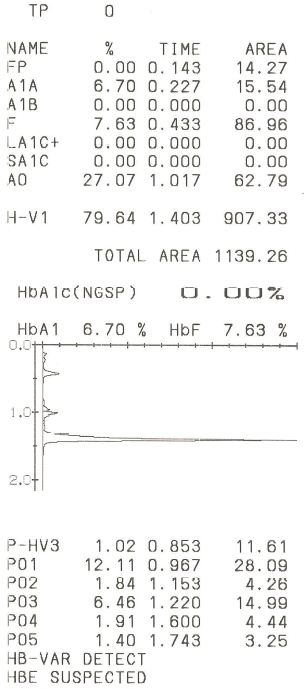


I)

H)


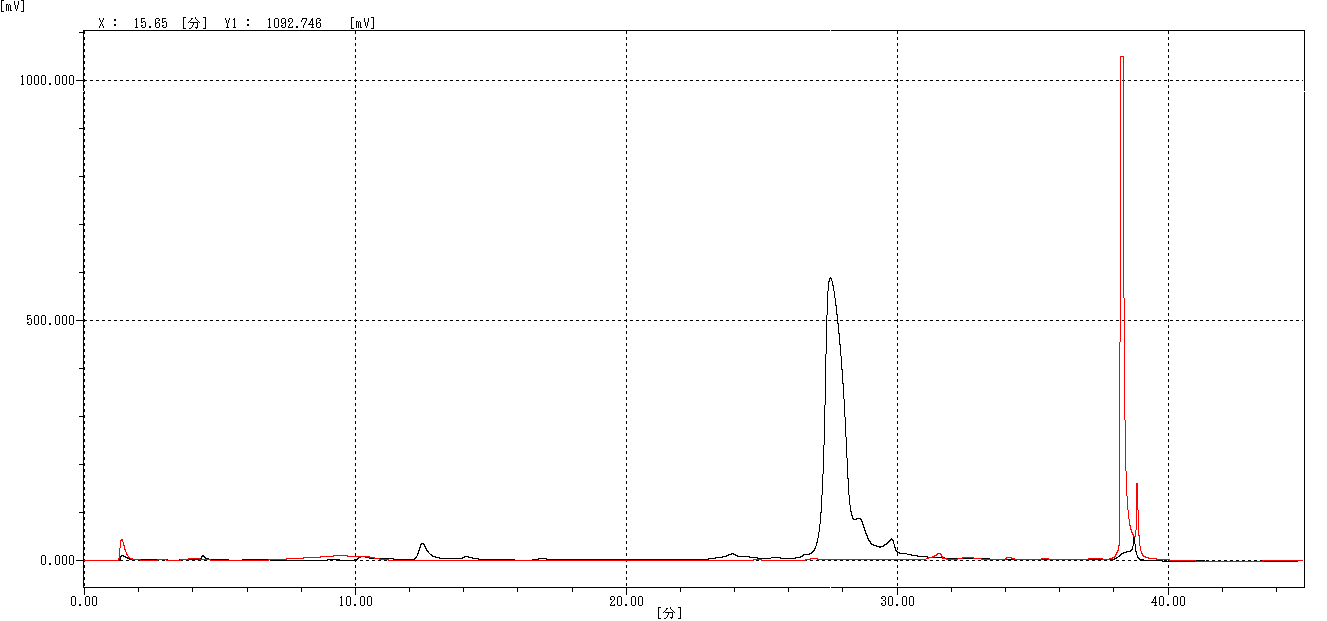

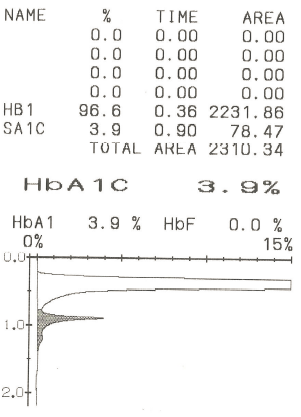


Supplemental Figure 5

Results of Hb S (homozygote or hemizygote) (Sample No. 600-13-9994-35).

(A) Results of the globin gene analysis. The mutation was identified at position 6 [from GAG (Glu) to GTG (Val)]. Chromatograms obtained using HPLC methods; (B) GR01-Short, (C) GR01-Long, (D) G11-Standard, (E) G11-Variant, (F) GX, (G) G8-Variant, and (H) G8-AF. (I) Chromatograms of HPLC system based on KO500 (Red: Variant sample, Black: control sample).


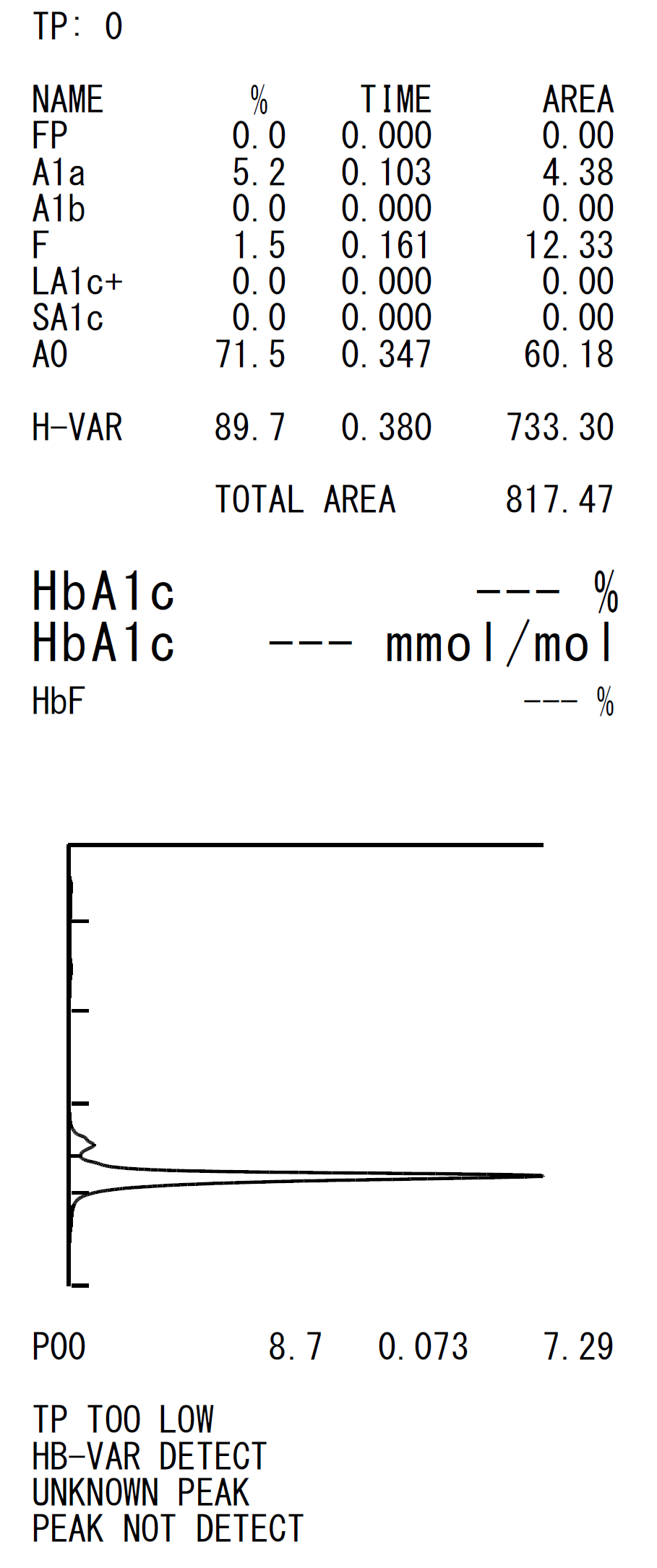

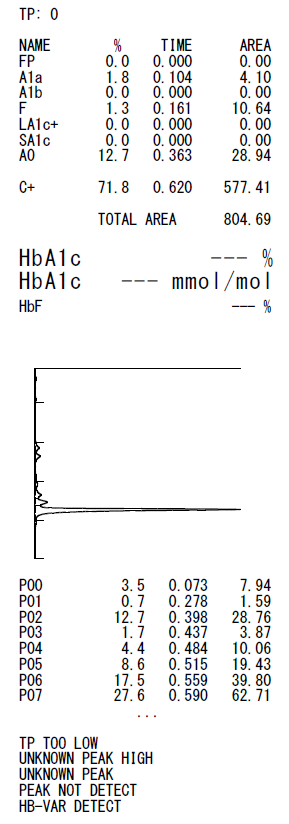


C)

B)

A)


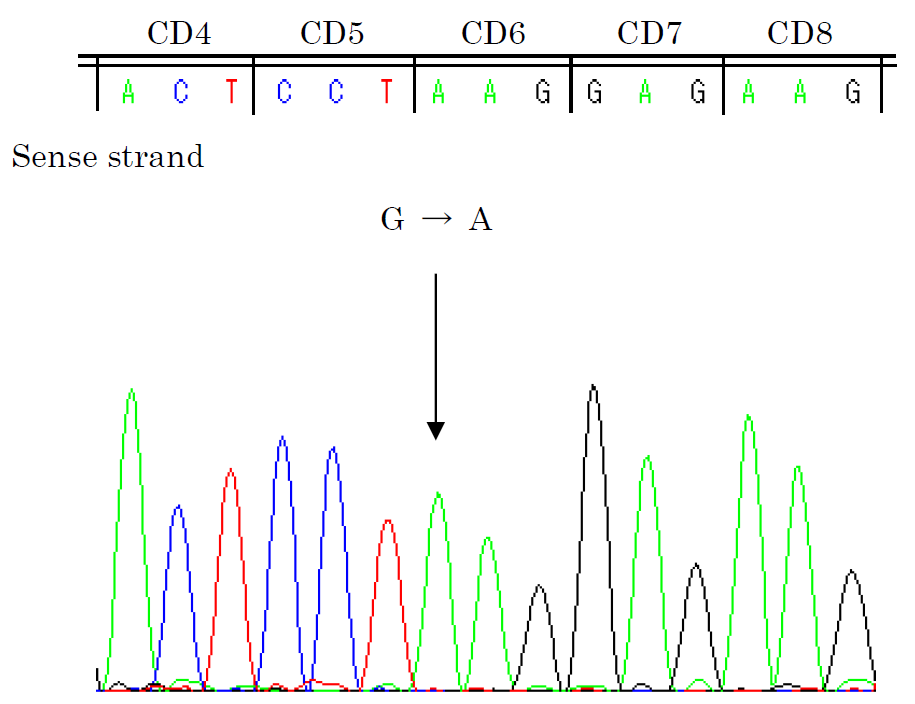


G)

D)

E)

F)


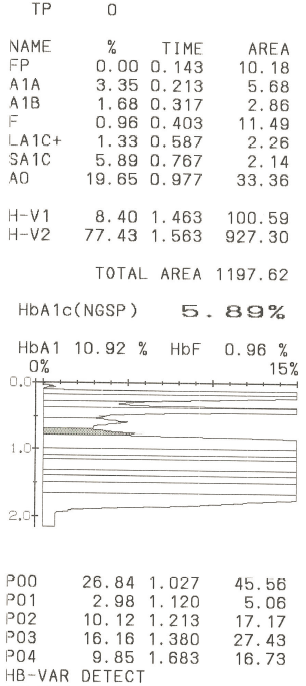

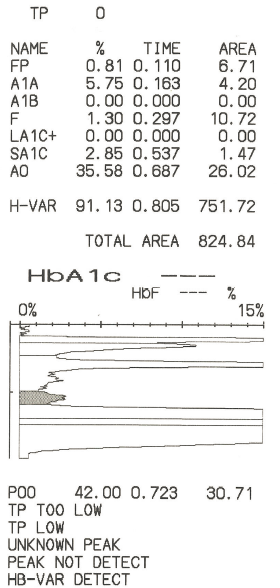

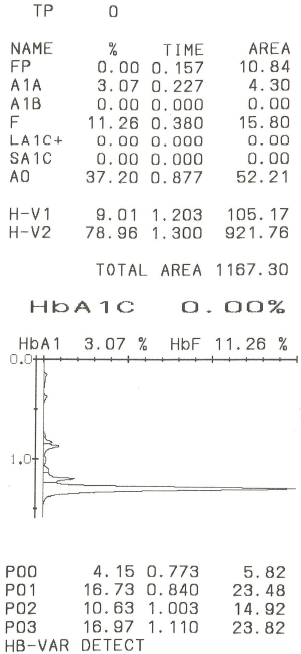

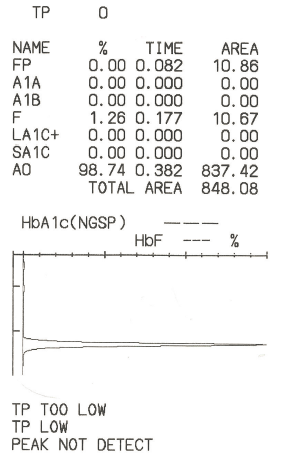


I)

H)


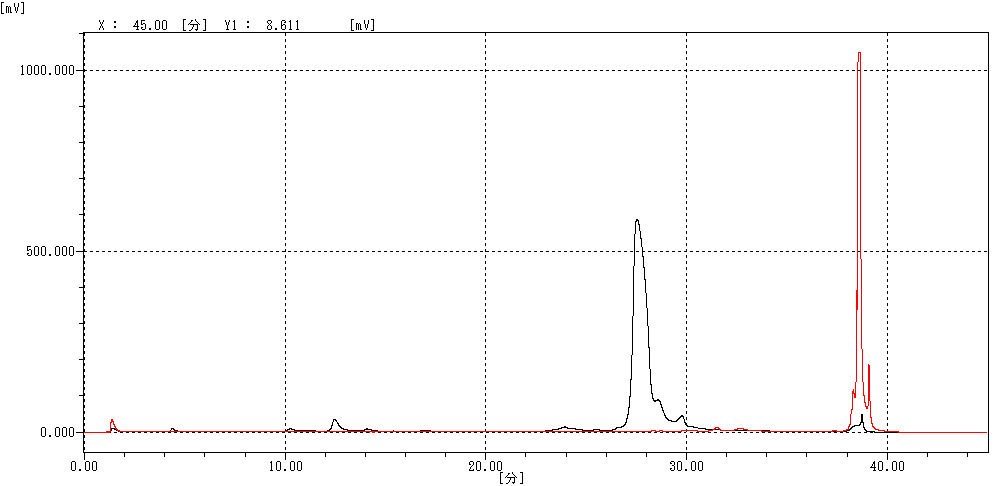

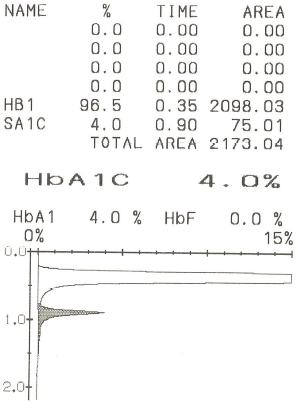


Supplemental Figure 6

Results of Hb C (homozygote or hemizygote) (Sample No. 600-13-9991-32).

(A) Results of the globin gene analysis. The mutation was identified at position 6 [from GAG (Glu) to AAG (Lys)]. Chromatograms obtained using HPLC methods; (B) GR01-Short, (C) GR01-Long, (D) G11-Standard, (E) G11-Variant, (F) GX, (G) G8-Variant, and (H) G8-AF. (I) Chromatograms of HPLC system based on KO500 (Red: Variant sample, Black: control sample).


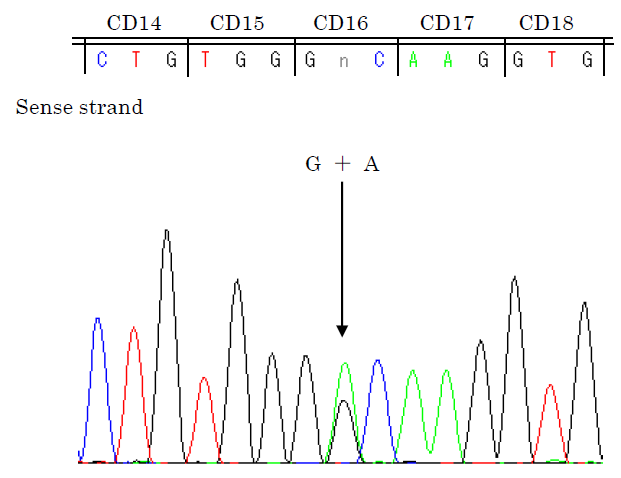


C)

B)

A)


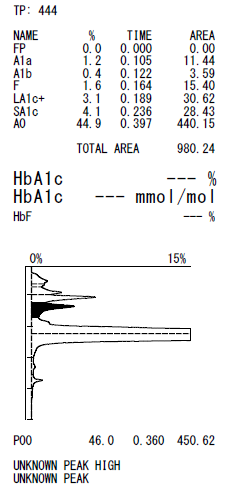

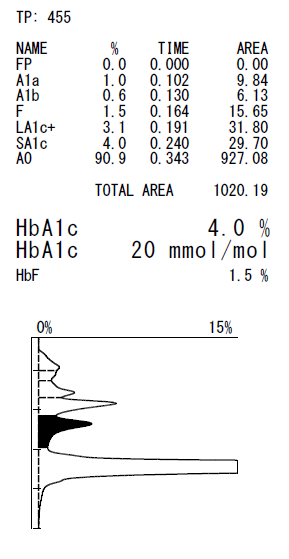


G)

D)

E)

F)


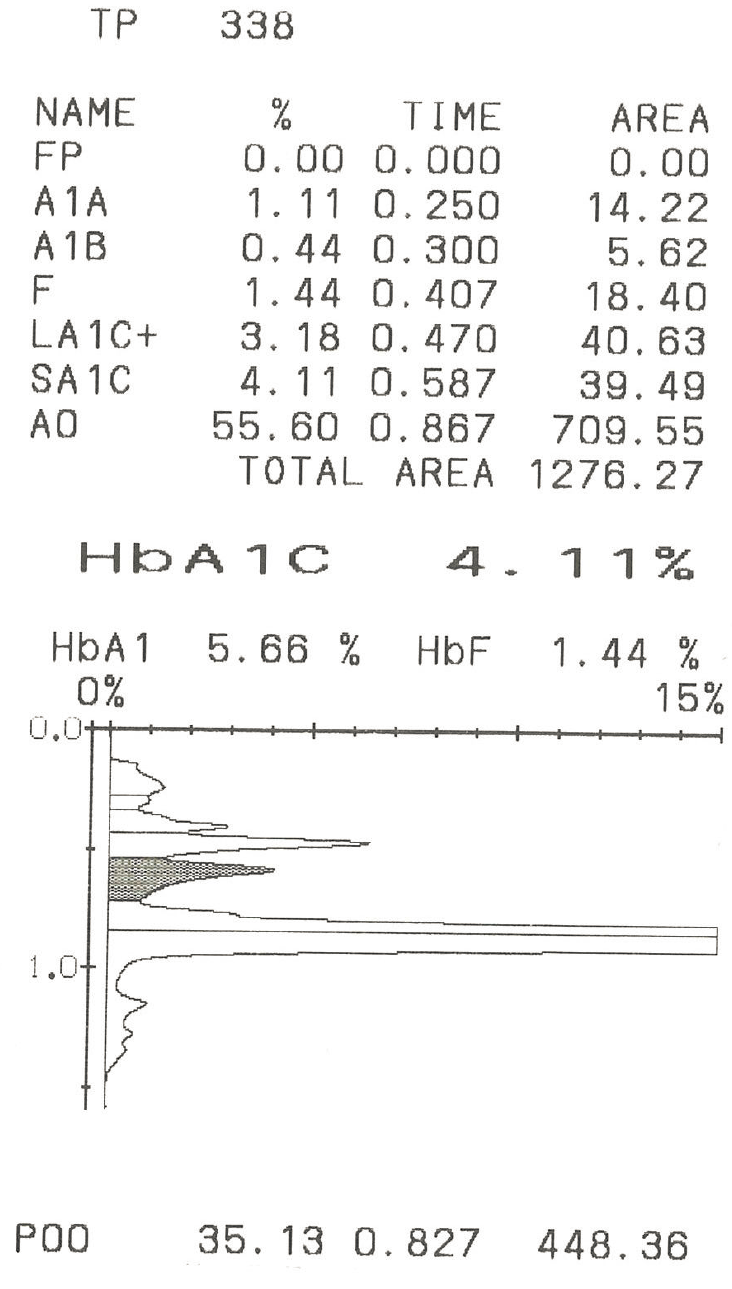

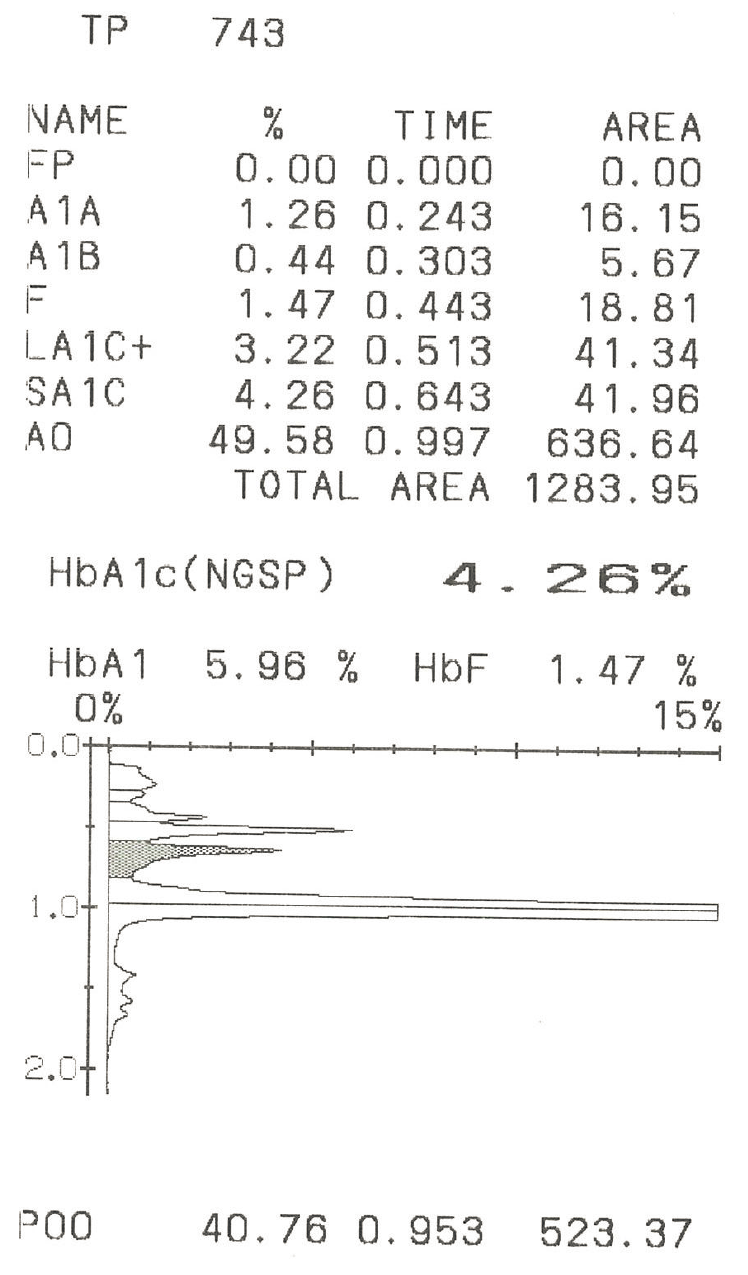

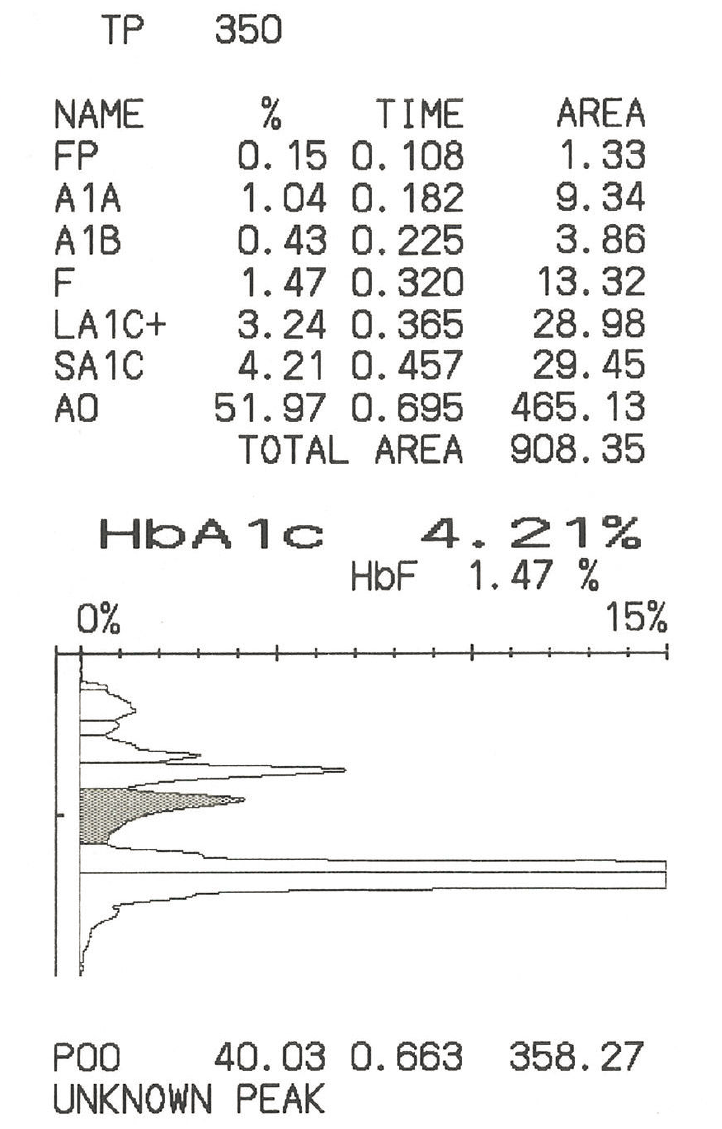

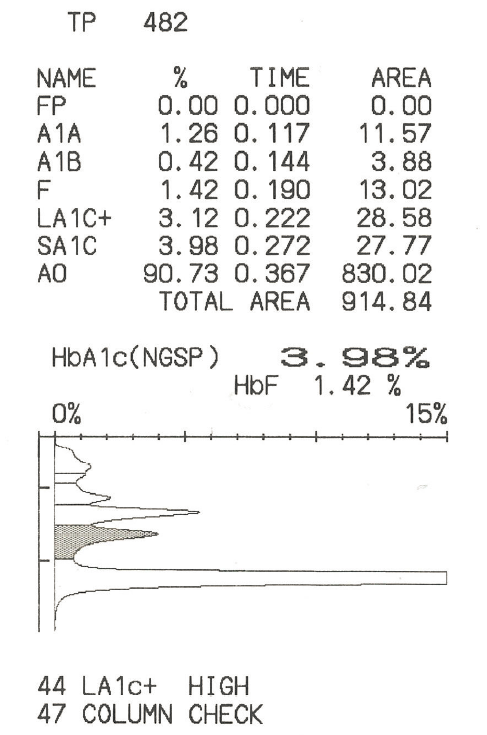


I)

H)


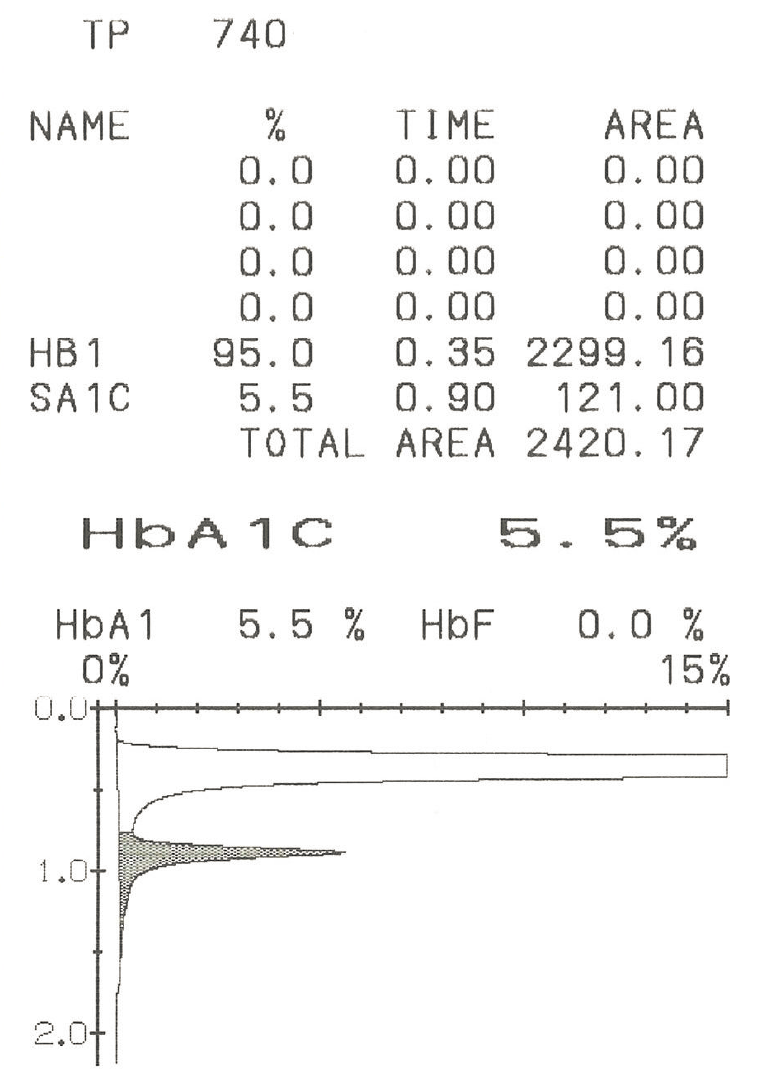

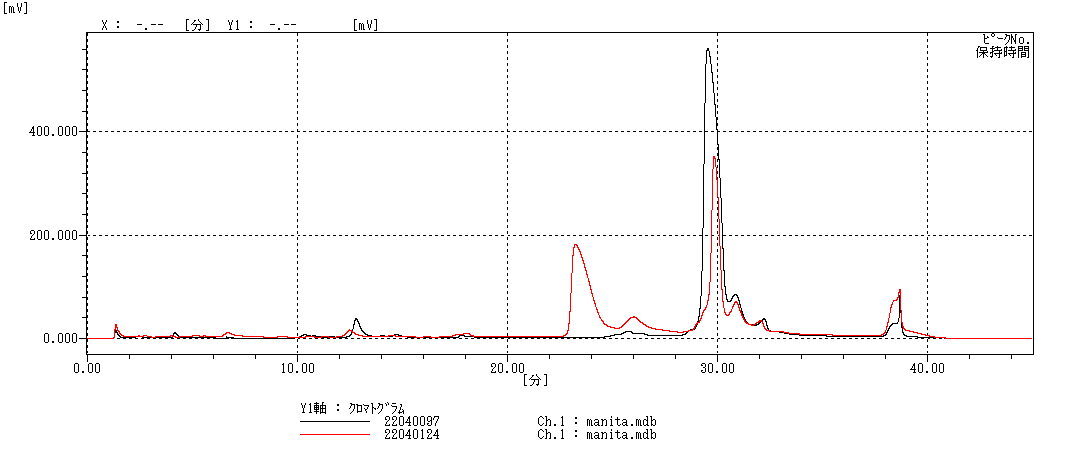


Supplemental Figure 7

Results of Hb J-Baltimore (heterozygote) (Sample No. 600-11-01309-6).

(A) Results of the globin gene analysis. The mutation was identified at position 16 [from GGC (Gly) to GAC (Asp)].

Chromatograms obtained using HPLC methods; GR01-Short (B), GR01-Long (C), G11-Standard (D), G11-Variant (E), GX (F), G8-Variant (G) and G8-AF (H). (I) Chromatograms of HPLC system based on KO500 (Red: Variant sample, Black: control sample).

C)

B)

A)


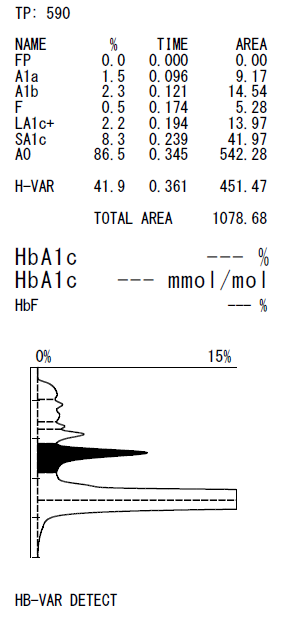

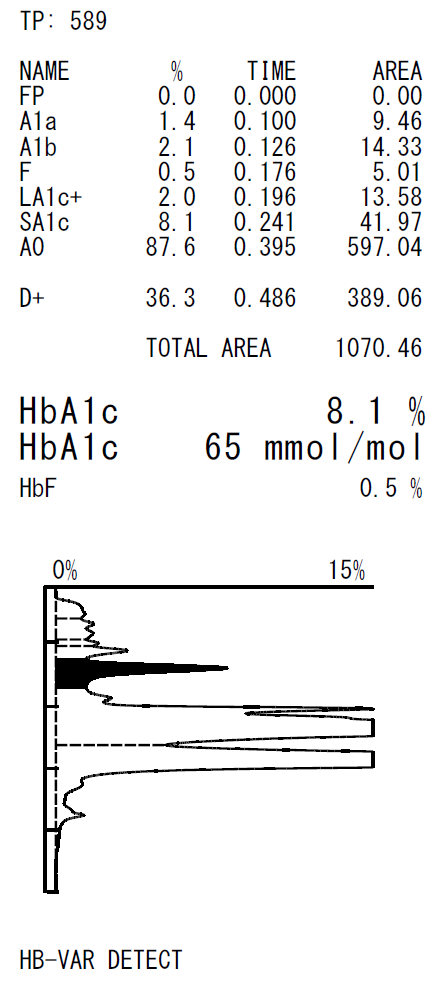

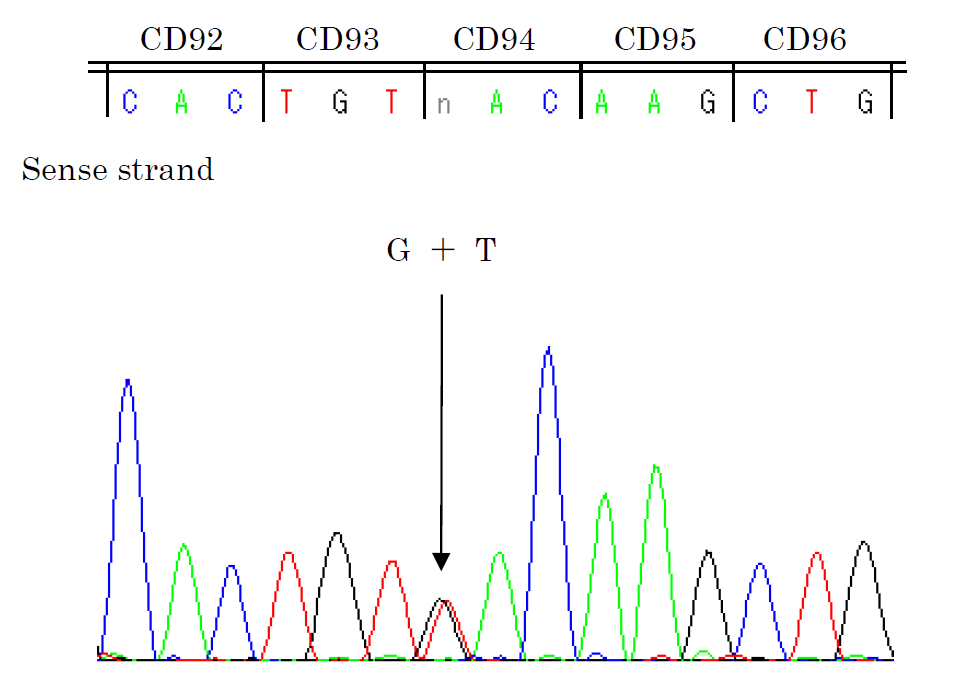


G)

D)

E)

F)


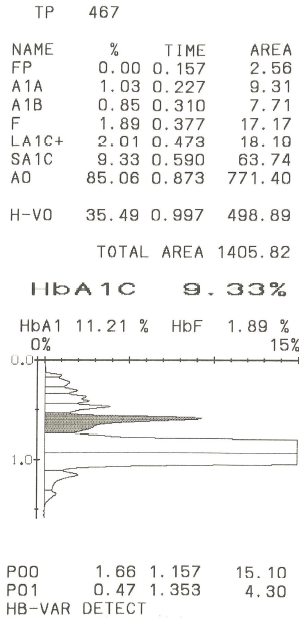

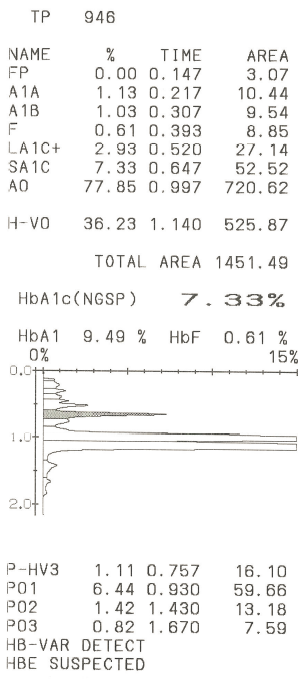

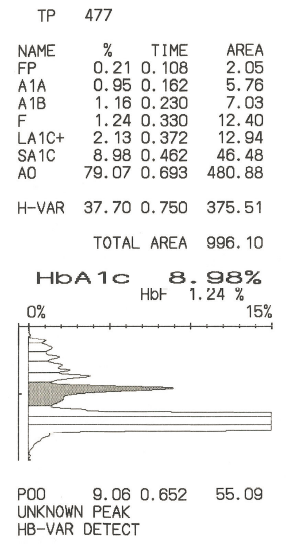

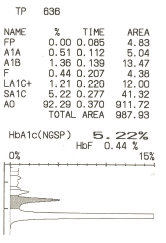


I)

H)


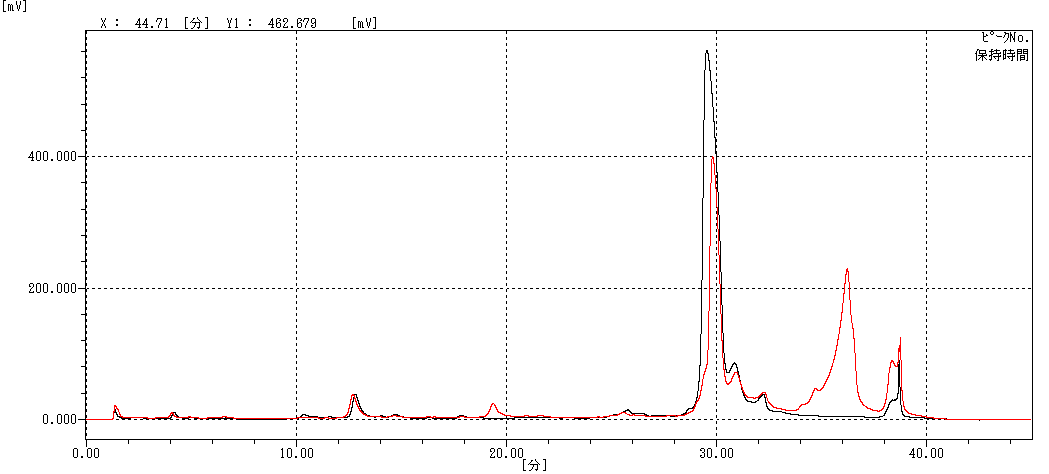

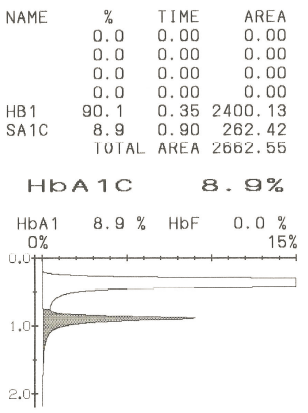


Supplemental Figure 8

Results of Hb Geldrop St Anna (heterozygote) (Sample No. 600-12-01301-17).

(A) Results of the globin gene analysis. The mutation was identified at position 94 [from GAC (Asp) to TAC (Tyr)]. Chromatograms obtained using HPLC methods; (B) GR01-Short, (C) GR01-Long, (D) G11-Standard, (E) G11-Variant, (F) GX, (G) G8-Variant, and (H) G8-AF. (I) Chromatograms of HPLC system based on KO500 (Red: Variant sample, Black: control sample).


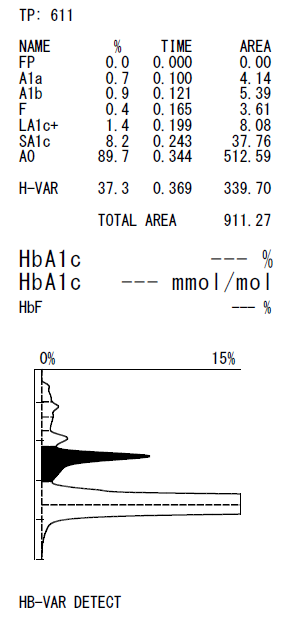

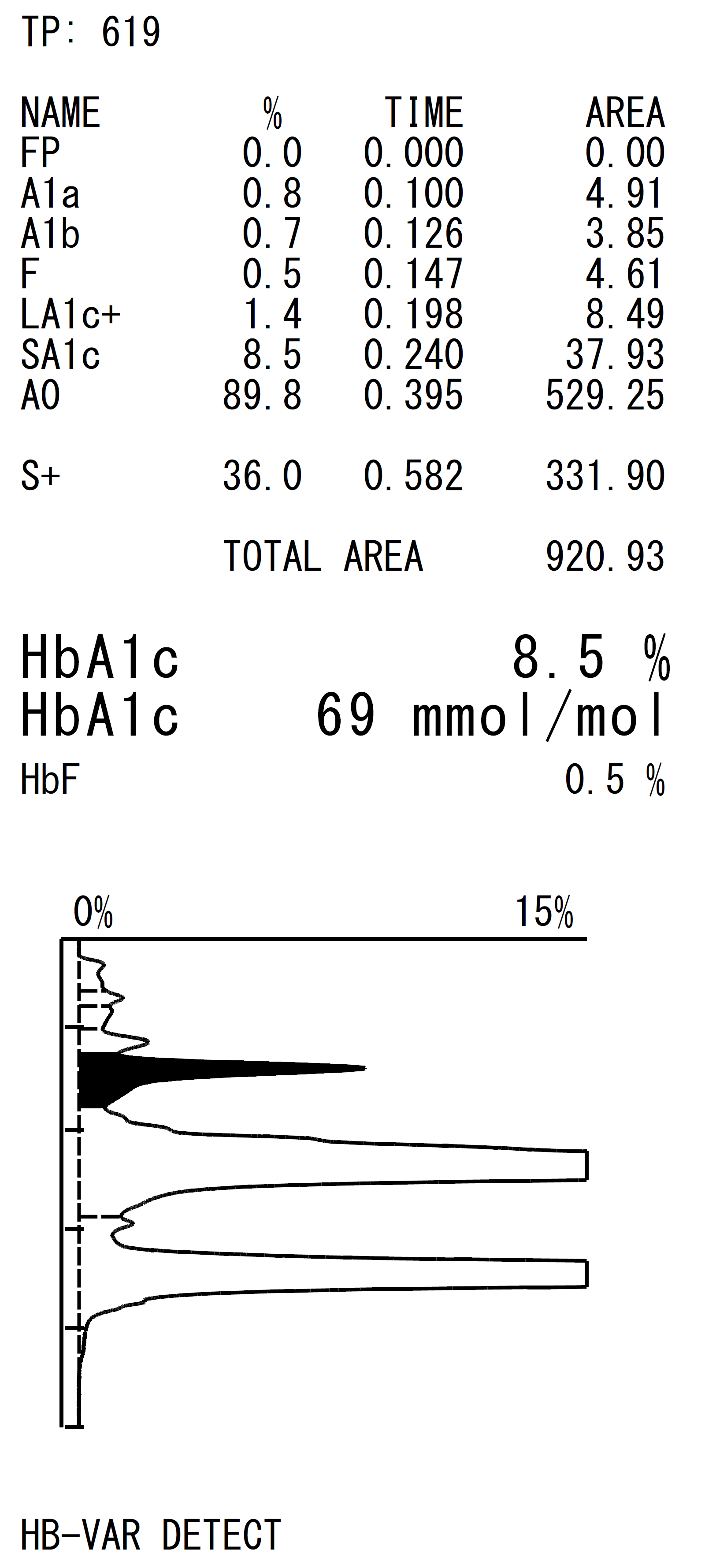


C)

B)

A)

G)

D)

E)

F)

I)

H)


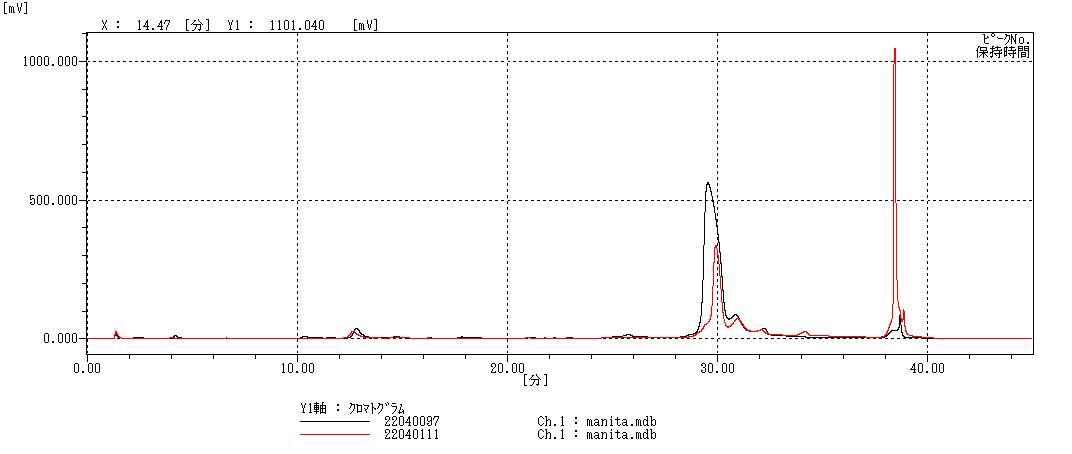


Supplemental Figure 9

Results of Hb O-Arab (heterozygote) (Sample No. 600-09-01329-12).

(A) Results of the globin gene analysis. The mutation was identified at position 121 [from GAA (Glu) to AAA (Lys)], and anti 3.7(+). Chromatograms obtained using HPLC methods; GR01-Short (B), GR01-Long (C), G11-Standard (D), G11-Variant (E), GX (F), G8-Variant (G) and G8-AF (H). (I) Chromatograms of HPLC system based on KO500 (Red: Variant sample, Black: control sample).

C)

B)

A)


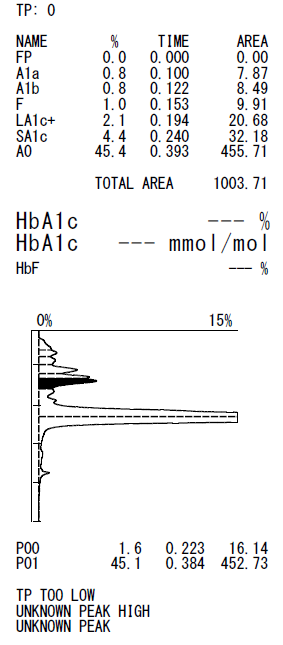

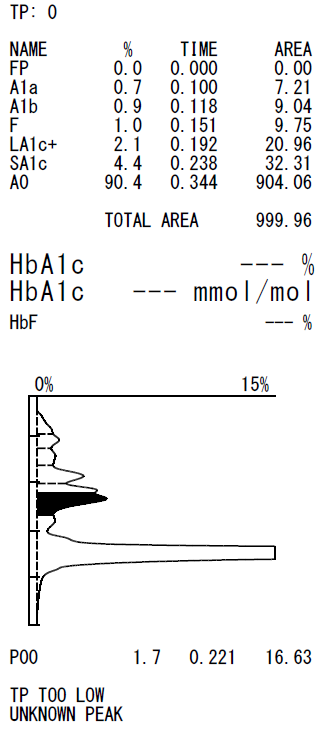

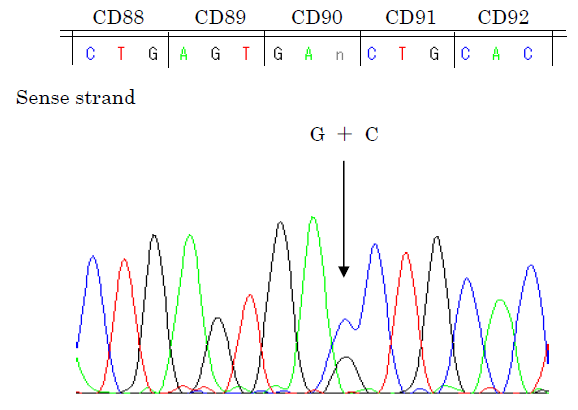


G)

D)

E)

F)


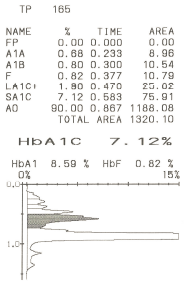

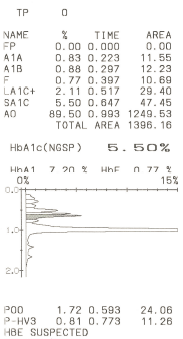

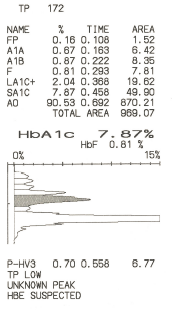

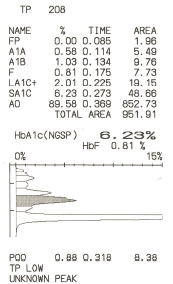


I)

H)


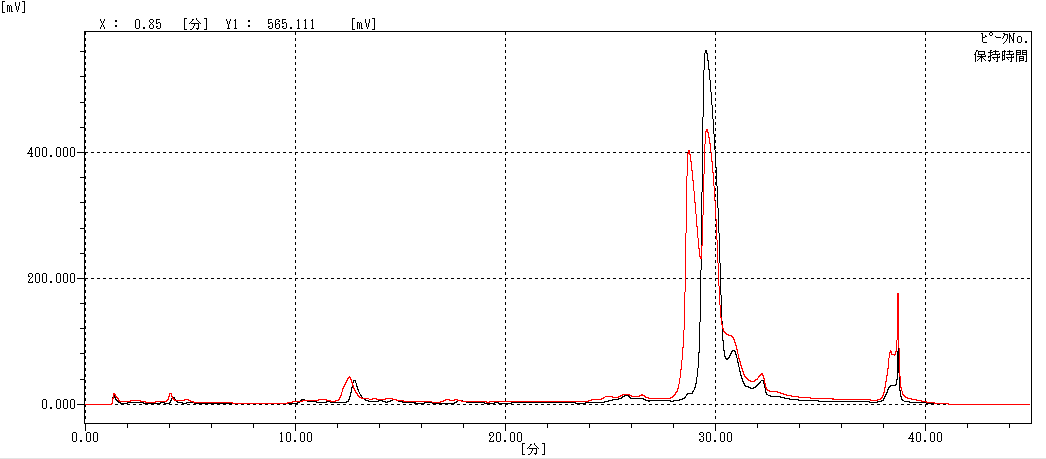

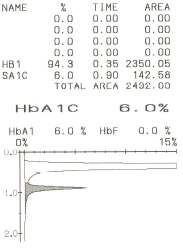


Supplemental Figure 10

Results of Hb Pierre-Bénite (heterozygote) (Sample No. 600-12-01302-18).

(A) Results of the globin gene analysis. The mutation was identified at position 90 [from GAG (Glu) to GAC (Asp)]. Chromatograms obtained using HPLC methods; (B) GR01-Short, (C) GR01-Long, (D) G11-Standard, (E) G11-Variant, (F) GX, (G) G8-Variant, and (H) G8-AF. (I) Chromatograms of HPLC system based on KO500 (Red: Variant sample, Black: control sample).

C)

B)

A)


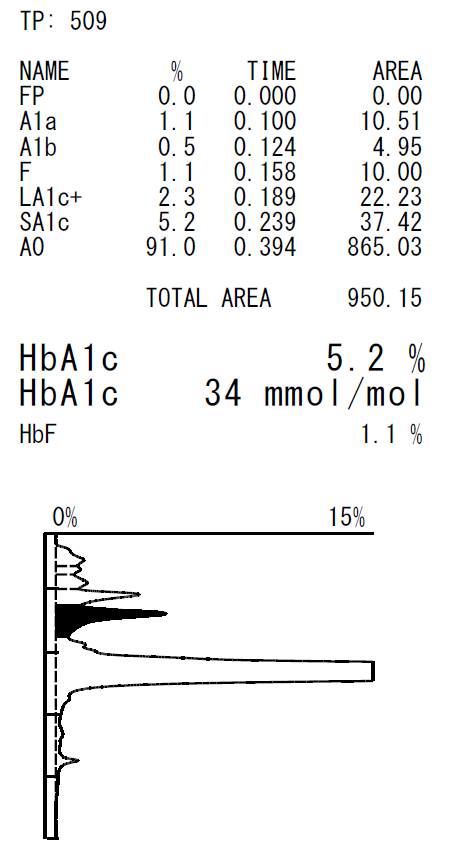

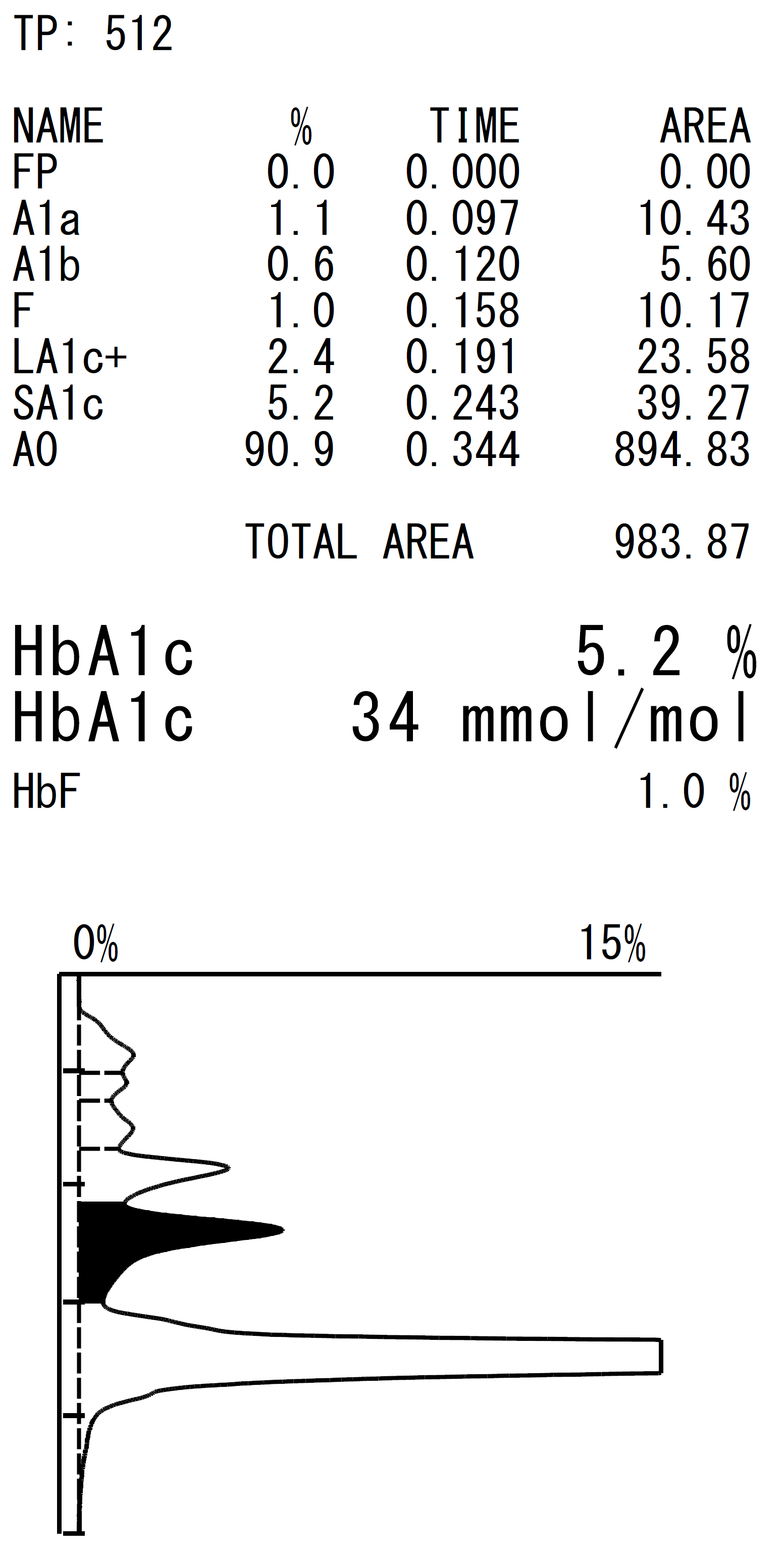


G)

D)

E)

F)

I)

H)


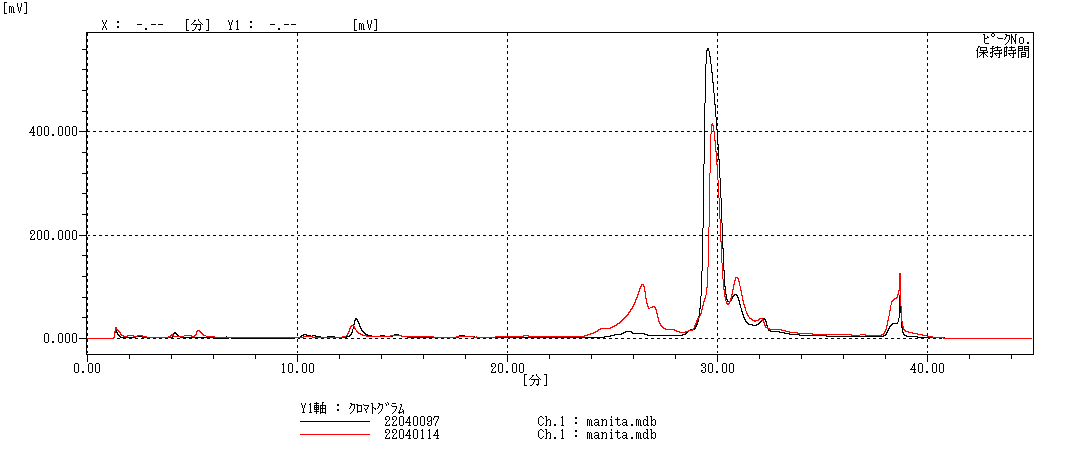


Supplemental Figure 11

Results of Hb Gouda (heterozygote) (Sample No. 600-09-01327-10).

(A) Results of the globin gene analysis. The mutation was identified at position 72 [from CAC (His) to CAA (Gln)].

Chromatograms obtained using HPLC methods; GR01-Short (B), GR01-Long (C), G11-Standard (D), G11-Variant (E), GX (F), G8-Variant (G) and G8-AF (H). (I) Chromatograms of HPLC system based on KO500 (Red: Variant sample, Black: control sample).

C)

B)

A)


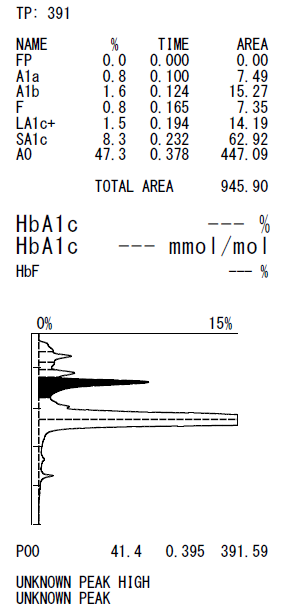

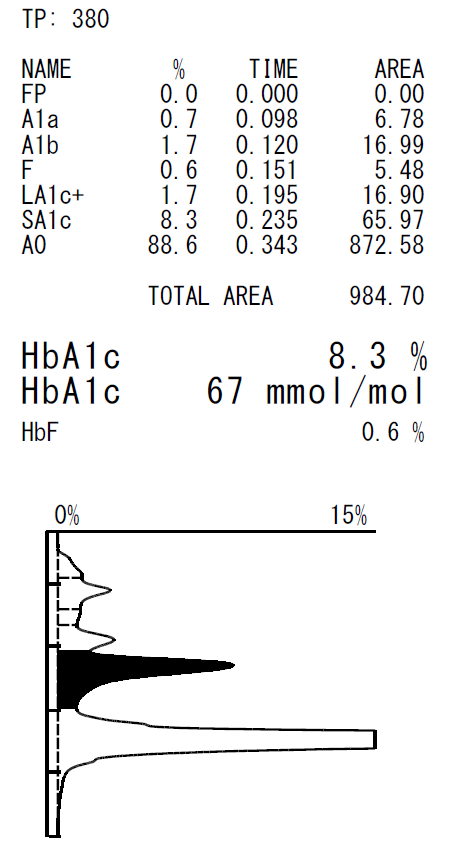

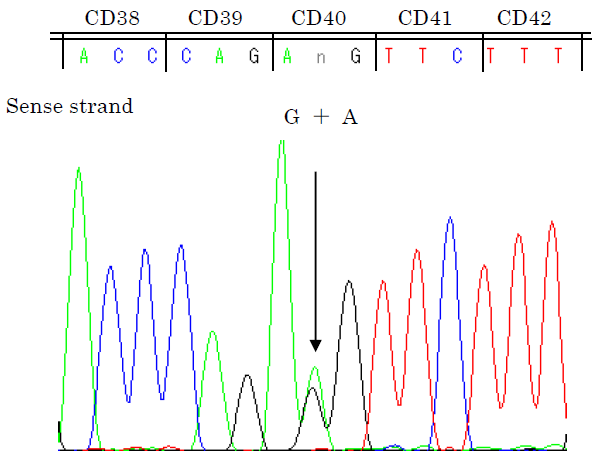


G)

D)

E)

F)

I)

H)


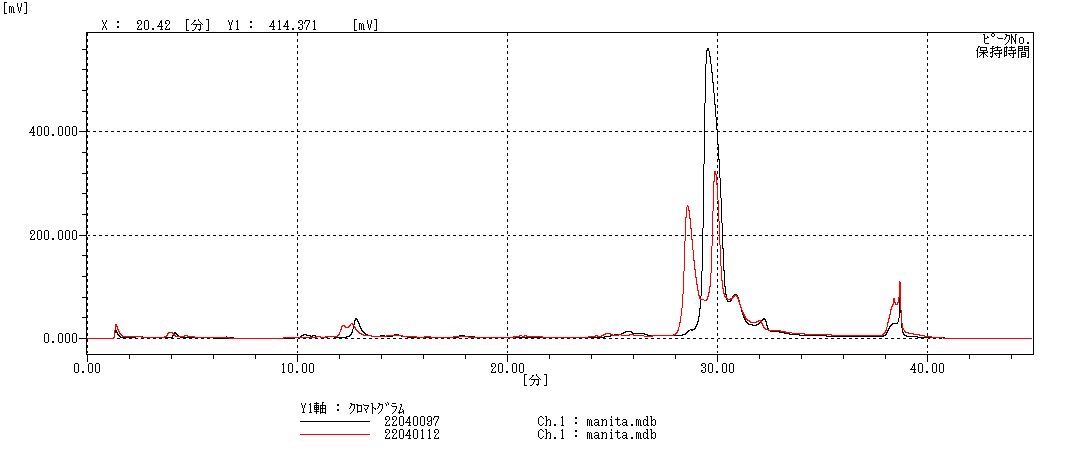


Supplemental Figure 12

Results of Hb Athens-GA (heterozygote) (Sample No. 600-11-01310-8).

(A) Results of the globin gene analysis. The mutation was identified at position 40 [from AGG (Arg) to AAG (Lys)].

Chromatograms obtained using HPLC methods; GR01-Short (B), GR01-Long (C), G11-Standard (D), G11-Variant (E), GX (F), G8-Variant (G) and G8-AF (H). (I) Chromatograms of HPLC system based on KO500 (Red: Variant sample, Black: control sample).

C)

B)

A)


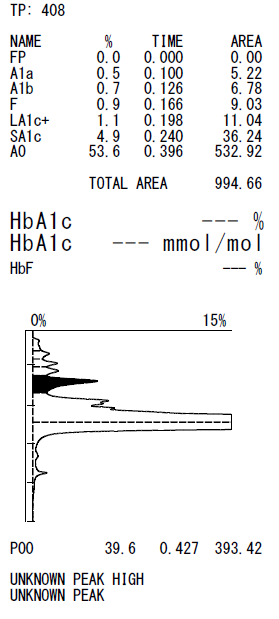

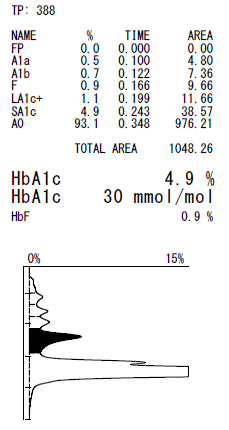

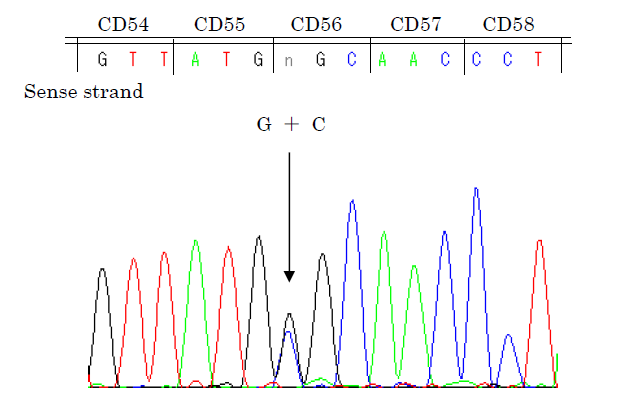


G)

D)

E)

F)


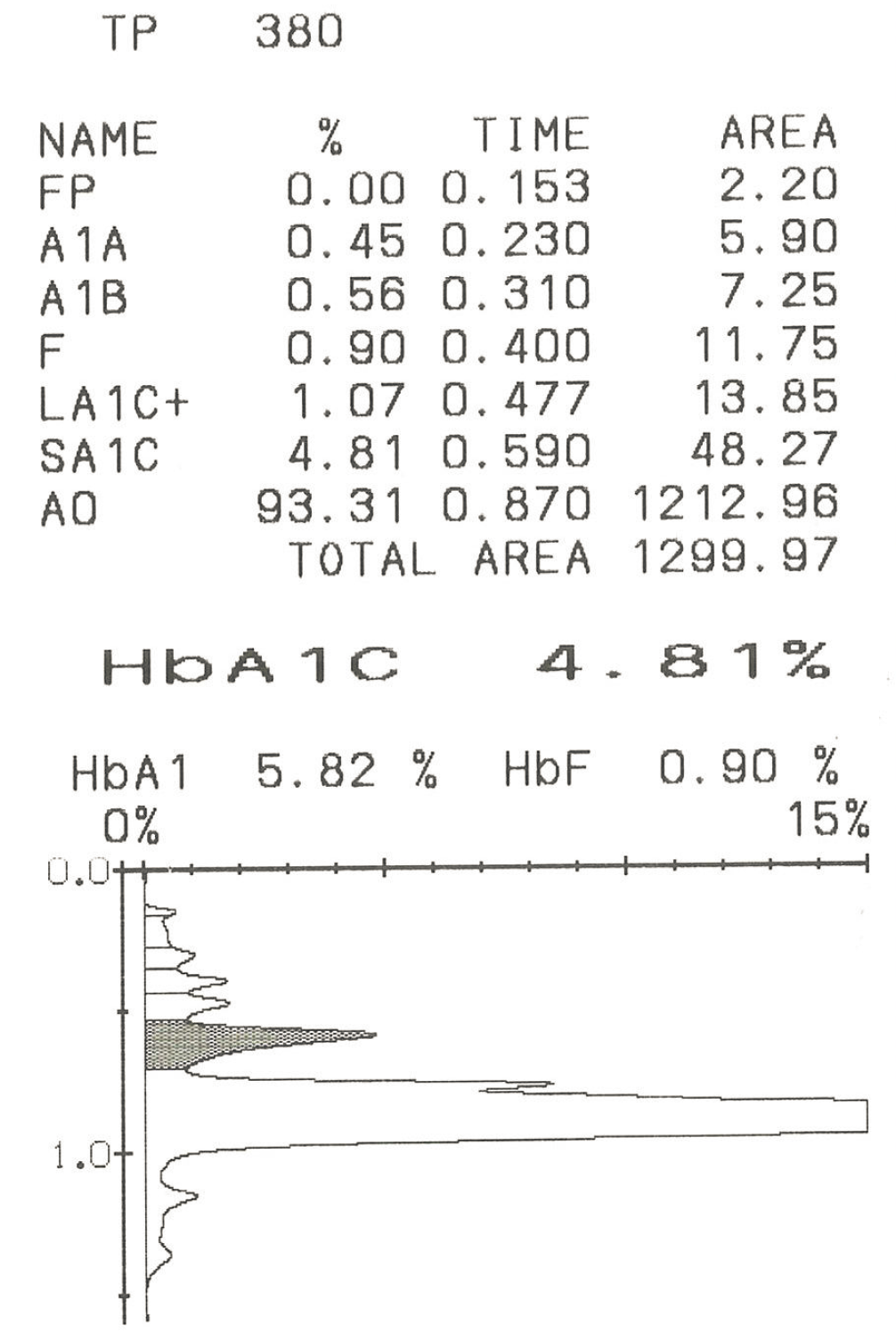

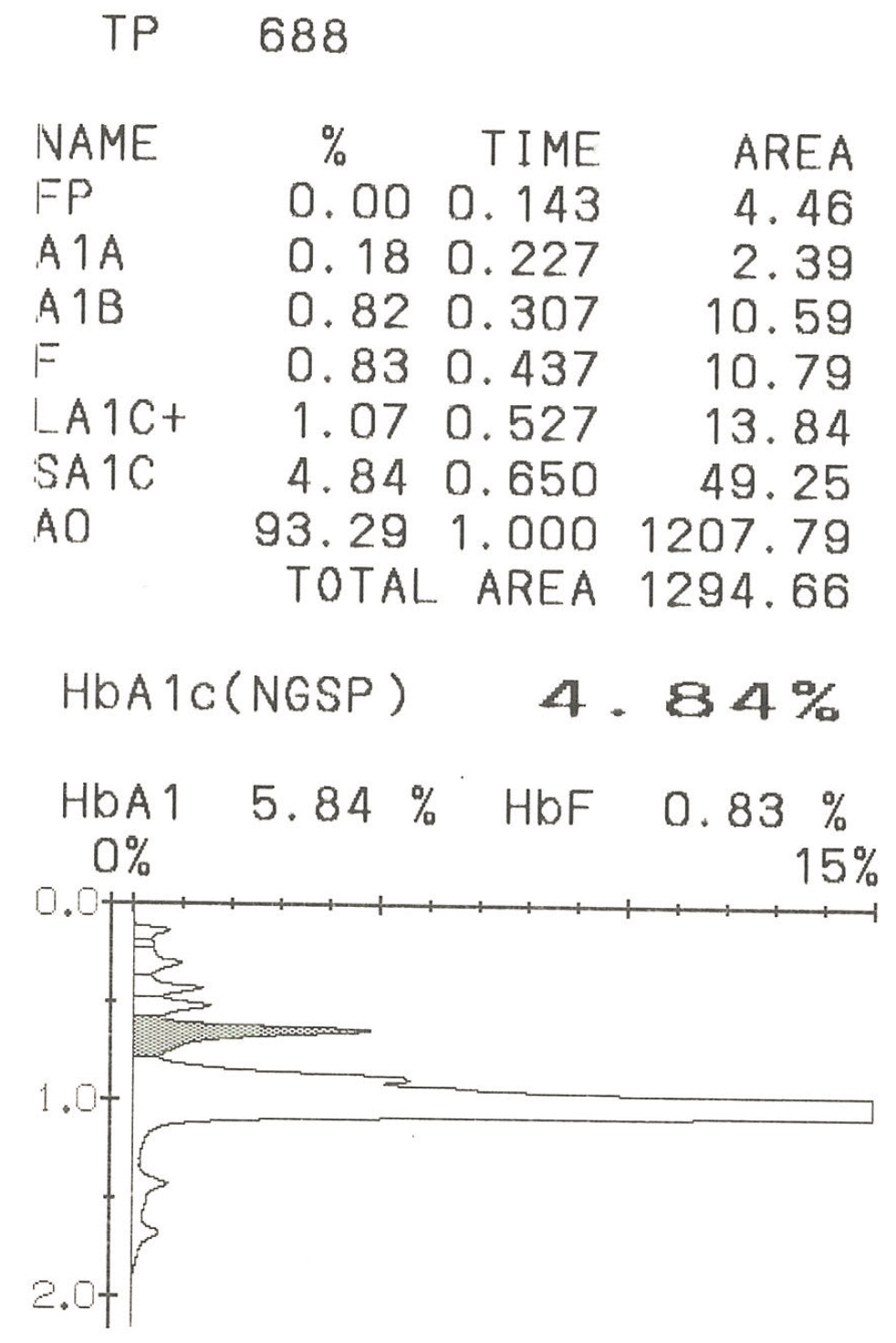

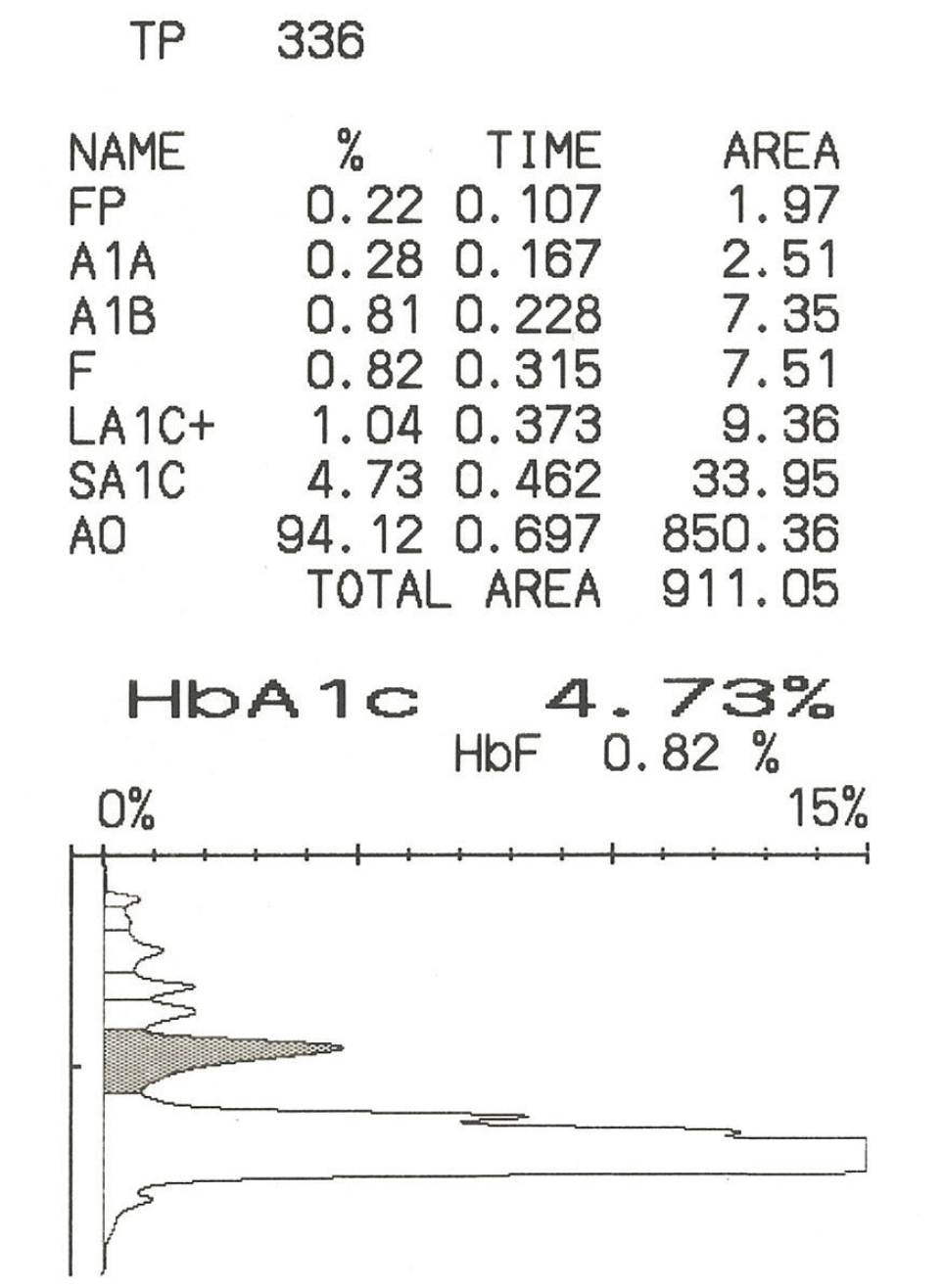

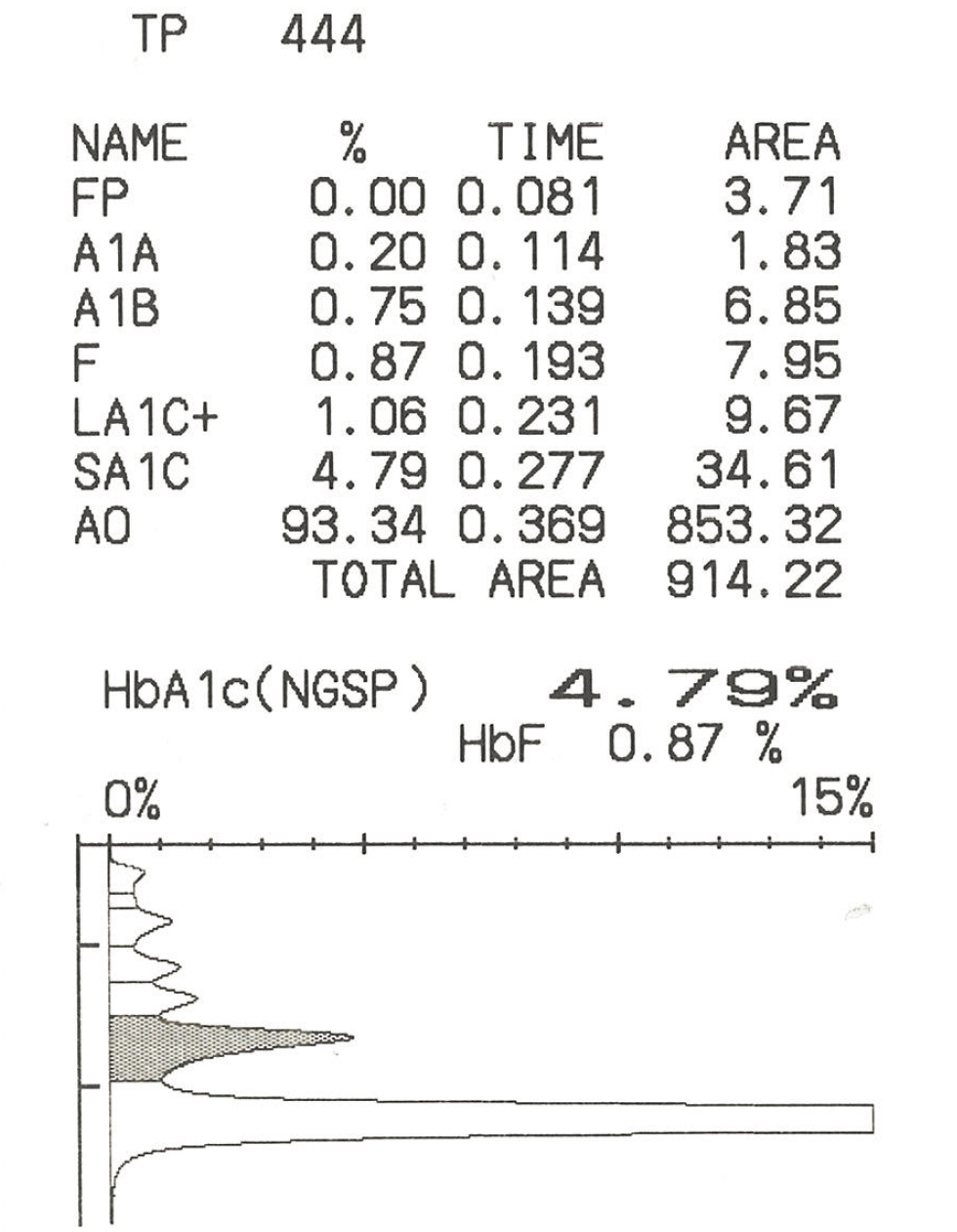


I)

H)


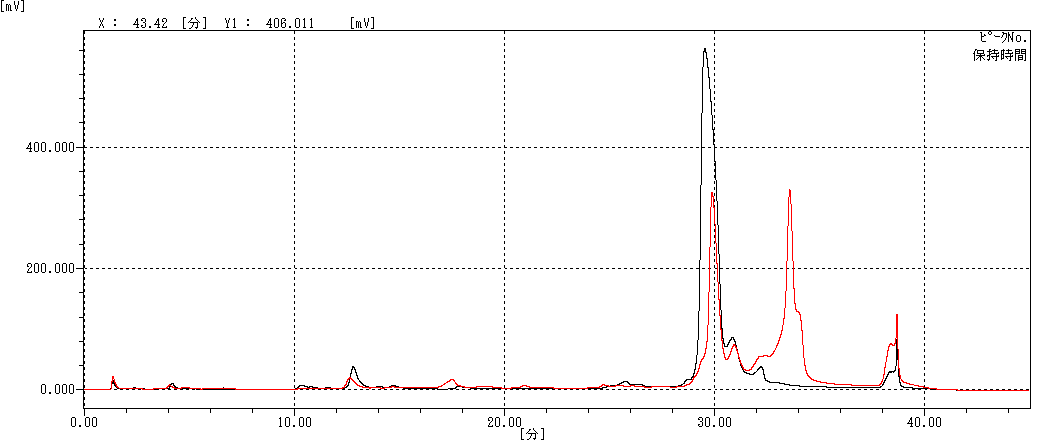


Supplemental Figure 13

Results of Hb Hamadan (heterozygote) (Sample No. 600-09-01326-9).

(A) Results of the globin gene analysis. The mutation was identified at position 56 [from GGC (Gly) to CGC (Arg)]. Chromatograms obtained using HPLC methods; (B) GR01-Short, (C) GR01-Long, (D) G11-Standard, (E) G11-Variant, (F) GX, (G) G8-Variant, and (H) G8-AF. (I) Chromatograms of HPLC system based on KO500 (Red: Variant sample, Black: control sample).

B)

C)

A)

G)

D)

E)

F)

I)

H)

Supplemental Figure 14

Results of Hb K-Ibadan (heterozygote) (Sample No. 600-09-01328-11).

(A) Results of the globin gene analysis. The mutation was identified at position 46 [from GGG (Gly) to GAG (Glu)].

Chromatograms obtained using HPLC methods; GR01-Short (B), GR01-Long (C), G11-Standard (D), G11-Variant (E), GX (F), G8-Variant (G) and G8-AF (H). (I) Chromatograms of HPLC system based on KO500 (Red: Variant sample, Black: control sample).

C)

B)

A)

G)

D)

E)

F)

I)

H)

Supplemental Figure 15

Results of Hb Ethiopia (heterozygote) (Sample No. 600-17-01304-23).

(A) Results of the globin gene analysis. The mutation was identified at position 140 [from TAC (Tyr) to CAC (His)]. Chromatograms obtained using HPLC methods; (B) GR01-Short, (C) GR01-Long, (D) G11-Standard, (E) G11-Variant, (F) GX, (G) G8-Variant, and (H) G8-AF. (I) Chromatograms of HPLC system based on KO500 (Red: Variant sample, Black: control sample).

C)

B)

A)

G)

D)

E)

F)

I)

H)

Supplemental Figure 16

Results of Hb Gorwihl (heterozygote) (Sample No. 600-11-01304-14).

(A) Results of the globin gene analysis. The mutation was identified at position 5 [from CTT (Pro) to GCT (Ala)]. Chromatograms obtained using HPLC methods; (B) GR01-Short, (C) GR01-Long, (D) G11-Standard, (E) G11-Variant, (F) GX, (G) G8-Variant, and (H) G8-AF. (I) Chromatograms of HPLC system based on KO500 (Red: Variant sample, Black: control sample).

C)

B)

A)

G)

D)

E)

F)

I)

H)

Supplemental Figure 17

Results of Hb Q-Iran (heterozygote) (Sample No. 600-11-01303-13).

(A) Results of the globin gene analysis. The mutation was identified at position 75 [from GAC (Asp) to CAC (His)]. Chromatograms obtained using HPLC methods; GR01-Short (B), GR01-Long (C), G11-Standard (D), G11-Variant (E), GX (F), G8-Variant (G) and G8-AF (H). (I) Chromatograms of HPLC system based on KO500 (Red: Variant sample, Black: control sample).

C)

B)

A)

G)

D)

E)

F)

I)

H)

Supplemental Figure 18

Results of Hb Köln (heterozygote) (Sample No. 600-11-01306-16).

(A) Results of the globin gene analysis. The mutation was identified at position 98 [from GTG (Val) to ATG (Met)]. Chromatograms obtained using HPLC methods; (B) GR01-Short, (C) GR01-Long, (D) G11-Standard, (E) G11-Variant, (F) GX, (G) G8-Variant, and (H) G8-AF. (I) Chromatograms of HPLC system based on KO500 (Red: Variant sample, Black: control sample).

C)

B)

A)

G)

D)

E)

F)

I)

H)

Supplemental Figure 19

Results of Hb Hounslow (heterozygote) (Sample No. 600-11-01305-15).

(A) Results of the globin gene analysis. The mutation was identified at position 80 [from AAC (Asn) to TAC (Tyr)]. Chromatograms obtained using HPLC methods; (B) GR01-Short, (C) GR01-Long, (D) G11-Standard, (E) G11-Variant, (F) GX, (G) G8-Variant, and (H) G8-AF. (I) Chromatograms of HPLC system based on KO500 (Red: Variant sample, Black: control sample).

C)

B)

A)

G)

D)

E)

F)

I)

H)

Supplemental Figure 20

Results of Hb Riccarton (heterozygote) (Sample No. 600-12-1312-25).

(A) Results of the globin gene analysis. The mutation was identified at position 51 [from GGC (Gly) to AGC (Ser)]. Chromatograms obtained using HPLC methods; (B) GR01-Short, (C) GR01-Long, (D) G11-Standard, (E) G11-Variant, (F) GX, (G) G8-Variant, and (H) G8-AF. (I) Chromatograms of HPLC system based on KO500 (Red: Variant sample, Black: control sample).

C)

B)

A)

G)

D)

E)

F)

I)

H)

Supplemental Figure 21

Results of Hb G-Accra (heterozygote) (Sample No. 600-12-1311-24).

(A) Results of the globin gene analysis. The mutation was identified at position 73 [from GAT (Asp) to AAT (Asn)]. Chromatograms obtained using HPLC methods; (B) GR01-Short, (C) GR01-Long, (D) G11-Standard, (E) G11-Variant, (F) GX, (G) G8-Variant, and (H) G8-AF. (I) Chromatograms of HPLC system based on KO500 (Red: Variant sample, Black: control sample).

C)

B)

A)

G)

D)

E)

F)

I)

H)

Supplemental Figure 22

Results of Hb Ullevaal (heterozygote) (Sample No. 600-19-0999-2).

(A) Results of the globin gene analysis. The mutation was identified at position 78 [from CTG (Leu) to CTG (Val)]. Chromatograms obtained using HPLC methods; (B) GR01-Short, (C) GR01-Long, (D) G11-Standard, (E) G11-Variant, (F) GX, (G) G8-Variant, and (H) G8-AF. (I) Chromatograms of HPLC system based on KO500 (Red: Variant sample, Black: control sample).

C)

B)

A)

G)

D)

E)

F)

I)

H)

Supplemental Figure 23

Results of Hb G-Philadelphia (heterozygote) (Sample No. 600-19-0999-3).

(A) Results of the globin gene analysis. The mutation was identified at position 68 [from AAC (Asn) to AAA (Lys)]. Chromatograms obtained using HPLC methods; (B) GR01-Short, (C) GR01-Long, (D) G11-Standard, (E) G11-Variant, (F) GX, (G) G8-Variant, and (H) G8-AF. (I) Chromatograms of HPLC system based on KO500 (Red: Variant sample, Black: control sample).

C)

B)

A)

G)

D)

E)

F)

I)

H)

Supplemental Figure 24

Results of Hb Melusine (heterozygote) (Sample No. 600-24-09999-1).

(A) Results of the globin gene analysis. The mutation was identified at position 114 [from CCC (Pro) to TCC (Ser)]. Chromatograms obtained using HPLC methods; (B) GR01-Short, (C) GR01-Long, (D) G11-Standard, (E) G11-Variant, (F) GX, (G) G8-Variant, and (H) G8-AF. (I) Chromatograms of HPLC system based on KO500 (Red: Variant sample, Black: control sample).

C)

B)

A)

G)

D)

E)

F)

I)

H)

Supplemental Figure 25

Results of β^+^-thalassemia (heterozygote) and Hb S (heterozygote) (Sample No. 600-17-01302-21).

(A) Results of the globin gene analysis. The mutation was identified at -88 [from C to T] and position 6 [from GAG (Glu) to GTG (Val)]; and -3.7(+). Chromatograms obtained using HPLC methods; (B) GR01-Short, (C) GR01-Long, (D) G11-Standard, (E) G11-Variant, (F) GX, (G) G8-Variant, and (H) G8-AF. (I) Chromatograms of HPLC system based on KO500 (Red: Variant sample, Black: control sample).

C)

B)

A)

G)

D)

E)

F)

I)

H)

Supplemental Figure 26

Results of β^0^-thalassemia (homozygote or hemizygote) (Sample No. 600-17-01303-22).

(A) Results of the globin gene analysis. The mutation was identified at position 5 (-CT) [from CCT (Pro) to C--]; and -3.7(+). Chromatograms obtained using HPLC methods; (B) GR01-Short, (C) GR01-Long, (D) G11-Standard, (E) G11-Variant, (F) GX, (G) G8-Variant, and (H) G8-AF. (I) Chromatograms of HPLC system based on KO500 (Red: Variant sample, Black: control sample).

C)

B)

A)

G)

D)

E)

F)

I)

H)

Supplemental Figure 27

Results of HbE (heterozygote) (Sample No. 600-017-9992-29).

(A) Results of the globin gene analysis. The mutation was identified at position 26 [from GAG (Glu) to AAG (Lys)]. Chromatograms obtained using HPLC methods; (B) GR01-Short, (C) GR01-Long, (D) G11-Standard, (E) G11-Variant, (F) GX, (G) G8-Variant, and (H) G8-AF. (I) Chromatograms of HPLC system based on KO500 (Red: Variant sample, Black: control sample).

A)

C)

B)

G)

D)

E)

F)

I)

H)

Supplemental Figure 28

Results of HbD-Los Angeles (heterozygote) (Sample No. 600-15-01301-1).

(A) Results of the globin gene analysis. The mutation was identified at position 121 [from GAA (Glu) to CAA (Gln)].

Chromatograms obtained using HPLC methods; GR01-Short (B), GR01-Long (C), G11-Standard (D), G11-Variant (E), GX (F), G8-Variant (G) and G8-AF (H). (I) Chromatograms of HPLC system based on KO500 (Red: Variant sample, Black: control sample).

C)

B)

A)

E)

G)

D)

F)

I)

H)

Supplemental Figure 29

Results of HbS (heterozygote) (Sample No. 600-17-9991-28).

(A) Results of the globin gene analysis. The mutation was identified at position 6 [from GAG (Glu) to GTG (Val)].

Chromatograms obtained using HPLC methods; GR01-Short (B), GR01-Long (C), G11-Standard (D), G11-Variant (E), GX (F), G8-Variant (G), and G8-AF (H). (I) Chromatograms of HPLC system based on KO500 (Red: Variant sample, Black: control sample).

B)

C)

A)

E)

G)

D)

F)

I)

H)

Supplemental Figure 30

Results of HbC (heterozygote) (Sample No. 600-17-9993-30).

(A) Results of the globin gene analysis. The mutation was identified at position 6 [from GAG (Glu) to AAG (Lys)].

Chromatograms obtained using HPLC methods; GR01-Short (B), GR01-Long (C), G11-Standard (D), G11-Variant (E), GX (F), G8-Variant (G), and G8-AF (H). (I) Chromatograms of HPLC system based on KO500 (Red: Variant sample, Black: control sample).
